# Production of hydrogen and carbon nanotubes from methane using a multi-pass floating catalyst chemical vapour deposition reactor with process gas recycling

**DOI:** 10.1038/s41560-025-01925-3

**Published:** 2025-12-01

**Authors:** Jack Peden, James Ryley, Jeronimo Terrones, Fiona Smail, James A. Elliott, Alan Windle, Adam Boies

**Affiliations:** 1 Department of Engineering, University of Cambridge, Cambridge, UK; 2 Q-Flo, Kent, UK; 3 Department of Materials Science and Metallurgy, University of Cambridge, Cambridge, UK; 4 Department of Mechanical Engineering, Stanford University, Stanford, CA USA

**Keywords:** Hydrogen energy, Carbon nanotubes and fullerenes, Synthesis and processing, Chemical engineering, Carbon capture and storage

## Abstract

Converting natural gas into hydrogen and solid carbon materials using methane pyrolysis presents a promising opportunity to produce sustainable fuels and materials. The production of hydrogen and bulk carbon nanotubes (CNTs) via methane pyrolysis has been demonstrated independently, but concurrent production from the same reactor has remained elusive. Here we present a multi-pass floating catalyst chemical vapour deposition (FCCVD) reactor that converts methane into hydrogen and CNT aerogel. Whereas previous FCCVD CNT production consumed hydrogen, the multi-pass reactor recycles the carrier gas to eliminate the need for a hydrogen input. This results in a net output of 85 vol% hydrogen alongside CNT aerogel and a 446-fold increase in molar process efficiency. Furthermore, the demonstrated use of biogas to produce CNT aerogel enables a potential net sequestration of CO_2_ from the atmosphere. The results of this study have been extrapolated to a pilot-scale reactor, using data gathered at a commercial facility, to consider the challenges and opportunities associated with scale-up.

## Abstract (web summary)

Methane pyrolysis produces hydrogen and carbon materials, but some approaches based on chemical vapour deposition actually consume hydrogen to mitigate unwanted side reactions. Here Peden et al. use gas recycling in a multi-pass floating catalyst chemical vapour deposition reactor to produce hydrogen alongside carbon nanotube aerogels.

## Main

Converting natural gas into hydrogen and solid carbon through methane pyrolysis presents a method to produce sustainable fuels and materials using hydrocarbon feedstocks that would otherwise be burned and produce greenhouse gas emissions. Hydrogen is sought as an energy vector for hard-to-electrify sectors and already plays a vital role as a precursor for artificial fertilizer production and other industries^1–3^. Today’s hydrogen production of ~100 Mt yr^−1^ contributes 2–3% of global annual greenhouse gas emissions^4,5^. Therefore, alternative, sustainable modes of production are needed. In addition, >10% of greenhouse gas emissions can be attributed to the production of materials, in particular, steel (7.2%) and concrete (3%)^6^. Alternative materials with reduced embedded emissions are also key to enabling the transition to a low greenhouse gas economy.

Methane pyrolysis is a process in which natural gas is decomposed into ‘turquoise’ hydrogen and solid carbon through the reaction

1 $${\rm{CH}}_{4}({\rm{g}})\to {\rm{C}}({\rm{s}})+2{\rm{H}}_{2}({\rm{g}}).$$

**Fig. 1:**
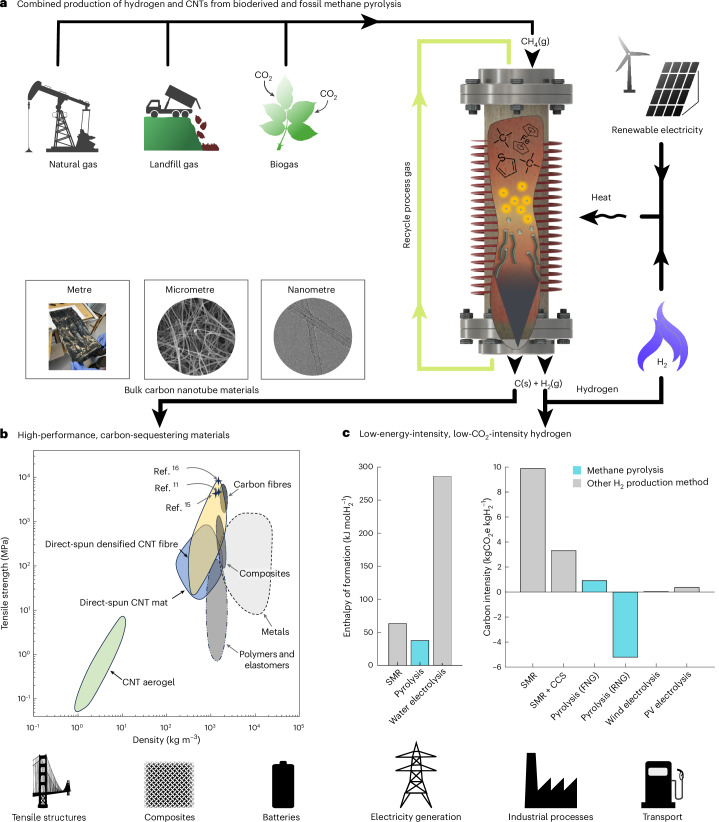
Methane pyrolysis to produce hydrogen and bulk CNT materials from methane. **a**, Methane (CH_4_(g)), obtained from natural gas, landfill gas or biogas, is decomposed inside a hot reactor into hydrogen gas (H_2_(g)) and solid carbon (C(s)), which is catalytically grown into carbon nanotubes and collected from the reactor as an aerogel. **b**, CNT materials possess exceptional tensile strength, finding applications in tensile and composite materials, as well as in additive markets such as batteries^11,15,16,47–49^. **c**, Hydrogen production by methane pyrolysis, using either fossil natural gas (FNG) or renewable natural gas (RNG), is energy efficient and has a low CO_2_ intensity compared to other hydrogen production technologies such as steam methane reforming (SMR), SMR with carbon capture and storage (SMR + CCS) and photovoltaic (PV) electrolysis, providing a clean fuel and precursor for electricity generation, industrial processes and transport^5,8,9^. Credit: icons in **a**–**c**, OpenClipart. Source data

The pyrolysis reaction is endothermic and endergonic (Δ*H*° = 37.4 kJ molH_2_^−1^ and Δ*G*° = 25.4 kJ molH_2_^−1^)^7^, yet requires less energy than hydrogen production by water splitting (Δ*H*° = 286 kJ molH_2_^−1^) or steam methane reforming (Δ*H*° = 63 kJ molH_2_^−1^)^8,9^ (Fig. 1c) with the added benefit of producing solid carbon that can serve as a useful material rather than CO_2_, which requires further energy for capture and storage. The solid carbon can take the form of graphite or carbon nanomaterials, which provide a material revenue stream^10^. Bulk carbon nanotube (CNT) materials are exceptionally versatile^11^, with fibres possessing high electrical conductivity (5 MS m^−1^)^12^, thermal conductivity (770 W m^−1^ K^−1^)^13^ and low density^14^ (1–2 kg m^−3^). CNT fibres have undergone a doubling of strength every 3 years^15^, culminating in the production in 2024 of a fibre with the highest recorded tensile strength of over 8 GPa (Fig. 1b)^16^. Methane pyrolysis can be CO_2_-negative if bioderived methane is used, taking atmospheric carbon sequestered by photosynthesis and converting it into solid materials^5^ (Fig. 1c). Methane pyrolysis, outlined in Fig. 1, thus provides an appealing technology to produce low-CO_2_-intensity and low-energy-intensity hydrogen alongside functional carbon materials that sequester rather than release carbon^5,17,18^.

The direct production of CNT mats and fibres from methane has been achieved using floating catalyst chemical vapour deposition (FCCVD) reactors^19^. CNT production using this process has hitherto consumed hydrogen, in the form of dilution gas to suppress unwanted side reactions, rather than produced it^20^. Hydrogen and CNTs have been produced simultaneously in fluidized bed systems^21–24^, and at least one patent exists for such a system^25^. However, these systems produce CNT powders, not structural materials. The largest market for powdered carbon seems to be carbon black, but at 18 Mt yr^−1^ (ref. ^26^), this is 100 times less than the rate at which carbon is produced in the form of natural gas (1.9 Gt yr^−1^)^27^. Structural materials, such as steel with a market of 1.6 Gt yr^−1^, are among the few commodities used on the same scale as hydrocarbons and thus present the most viable markets into which pyrolytic carbon materials could be absorbed^27^.

In this study, we successfully demonstrated the co-production of turquoise hydrogen and CNT mats using a multi-pass FCCVD reactor that recycles process gas, removing the need for an exogenous hydrogen supply during steady-state operation. We further found that this multi-pass process is suitable for both pure methane and methane contaminated with 33 vol% CO_2_. The latter simulates unrefined bioderived methane such as biogas or landfill gas^28^, which unlocks the potential for a carbon-negative process^5^. We investigated the differences between the multi-pass reactor and the traditional single-pass reactor in terms of process efficiencies and mass conversion, and characterized the resulting CNTs. We then applied our findings to data obtained from a pilot-scale reactor operated by industrial CNT producers to extrapolate the features of the multi-pass process to larger reactors.

### Multi-pass reactor configuration

**Fig. 2:**
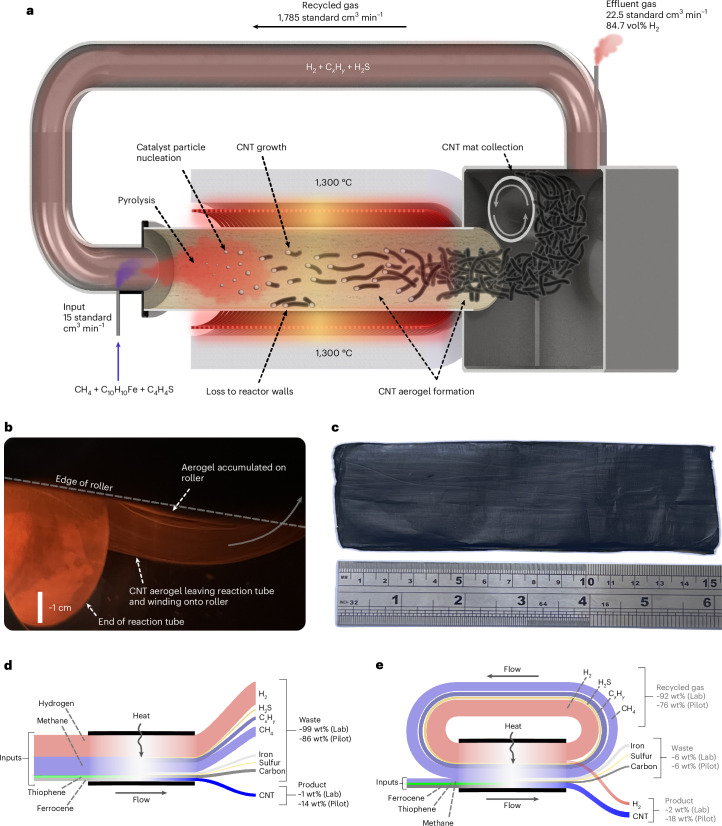
Configuration of the multi-pass reactor developed in this study. **a**, Schematic of the multi-pass reactor, showing ~99 vol% recycled process gas, CNT production and hydrogen effluent leaving the reactor. **b**, Photograph of the CNT aerogel leaving the reaction tube and winding onto the collection roller. **c**, Photograph of a mat made of multiple layers of CNT aerogel after removal from the reactor. **d**,**e**, Conceptual models showing mass flows in the single-pass (**d**) and multi-pass (**e**) processes. The multi-pass process is primarily composed of recycled gas passing repeatedly through the reactor in a loop, resulting in smaller input and waste streams. The magnitudes of the flows are not drawn to scale and the relative weight% of each stream is indicated for the lab-scale processes detailed in Fig. 4 and the pilot-scale processes detailed in Fig. 6. Source data

During steady-state operation, the multi-pass reactor is a quasi-closed loop with ~99 vol% of process gas circulating inside the reactor, making multiple passes of the high-temperature reaction zone. Relatively small quantities of precursors (methane and catalyst) are added to the reaction mixture before each pass of the furnace, producing CNT aerogel and hydrogen, which are removed from the reactor. Figure 2a shows a schematic of the multi-pass reactor operating in steady state, where 1,785 standard cm^3^ min^−1^ of process gas is recycled from the exhaust back to the injector. This gas consists primarily of hydrogen (H_2_), hydrocarbons (C_*x*_H_*y*_) and hydrogen sulfide (H_2_S). An additional 15 standard cm^3^ min^−1^ flow of methane and catalyst precursors (ferrocene (C_10_H_10_Fe) and thiophene (C_4_H_4_S)) is added to the recycled gas before injection into the furnace.

**Fig. 3:**
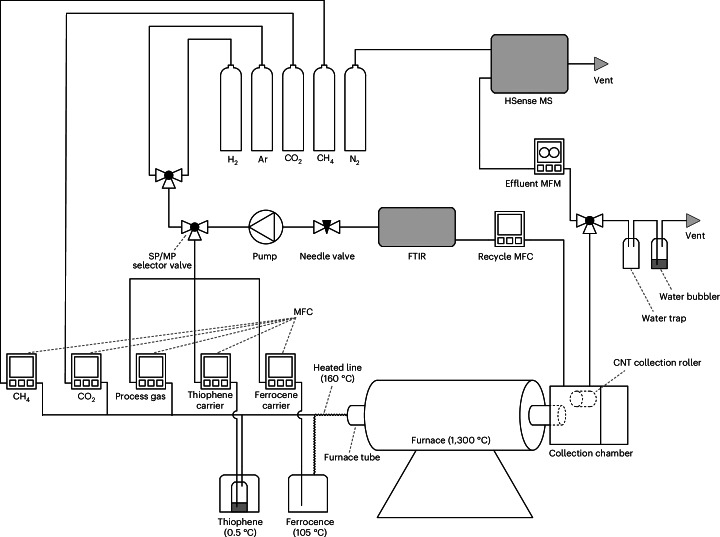
Schematic of the reactor used in this study. The same reactor can be operated in single-pass (SP) and multi-pass (MP) configurations, depending on the position of the SP/MP selector valve. The instruments used to characterize the process gas, including a Fourier transform infrared spectrometer (FTIR), HSense mass spectrometer (MS) and mass flow meter (MFM), are also shown. See Methods for more detail. MFC, mass flow controller.

Flowing through the furnace, the precursors are heated to ~1,300 °C, causing the methane to undergo pyrolysis to hydrogen and C_2_ species (primarily acetylene and ethylene) in the presence of nucleating iron–sulfur nanoparticles^29,30^. The catalyst particles act to nucleate and grow the CNTs^31^ from the pyrolysed species. These then agglomerate into bundles and form an interconnected aerogel^32,33^ that is extracted from the gas stream onto a rotating roller within the collection chamber. Figure 2b shows the aerogel exiting the reaction tube and being wound onto the roller, which collects the CNTs as a mat (Fig. 2c) or fibre. The process gases leave the collection chamber and 1,785 standard cm^3^ min^−1^ of the gas is recycled back to the injector for another pass of the reactor. An effluent flow of 22.5 standard cm^3^ min^−1^ containing 84.7 vol% H_2_ was measured during steady-state operation, corresponding to 19.1 standard cm^3^ min^−1^ of H_2_ production and 54% hydrogen production efficiency. The hydrogen concentration could be increased with pressure swing absorption (Supplementary Fig. 1), as is done in steam methane reforming processes^9^ (see Supplementary Note 1 and Supplementary Fig. 2 for details on how the steady state is achieved and Supplementary Fig. 3 and Supplementary Note 2 for more details on steady-state hydrogen production). Figure 3 shows a detailed schematic of the reactor used in this study and Supplementary Fig. 4 shows a photograph of the reactor.

In addition to enabling the combined production of CNTs and turquoise hydrogen, the multi-pass reactor proffers improved efficiency compared with a traditional single-pass reactor (Fig. 2d). In a single-pass system, reactants exit the reactor after a single pass of the furnace, resulting in a waste stream comprising unreacted gases and solid losses that accounts for 99 wt% of the mass throughput at the lab scale. The multi-pass process (Fig. 2e) reduces the size of the waste stream to 6 wt% by recycling 92 wt% of the total mass flow. By using recycled hydrogen in the multi-pass process, the need for an exogenous hydrogen supply is completely removed, resulting in a much smaller input stream compared with in the single-pass reactor. By recycling rather than consuming dilution hydrogen, the hydrogen produced by pyrolysis in the multi-pass reactor can be collected as a secondary product stream. Overall, these developments lead to a 33-fold reduction in the waste/product ratio between the lab-scale single-pass and multi-pass processes, from 99:1 (single-pass) to 3:1 (multi-pass). Further improvements come with scale-up to a pilot process, as will be discussed later.

### Mass conversion in the lab-scale process

**Fig. 4:**
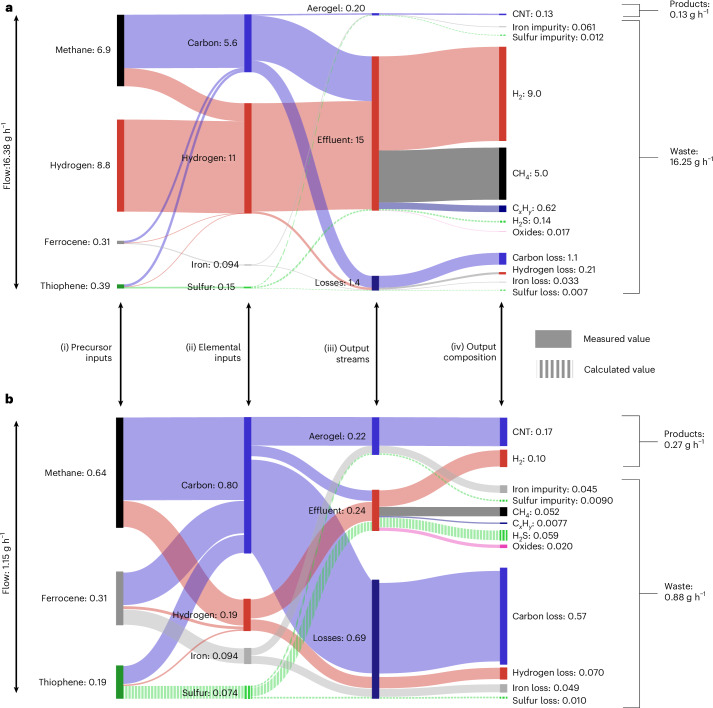
Mass conversion in the lab-scale processes. **a**,**b**, Mass conversion (g h^−1^) in the lab-scale single-pass (**a**) and multi-pass (**b**) processes. The Sankey diagrams represent the flow of mass into and out of the reactor. In the single-pass process (**a**), this represents all of the mass flowing through the reactor; in the multi-pass process (**b**), this represents only the mass added to and removed from the reactor, and does not include the large flow of gas that is continually recycled. The diagrams show the flow of mass from precursor inputs (i) on the left to elemental inputs (ii) and output streams (iii), as well as a breakdown of the chemical composition of output streams (iv) on the right. Solid flows indicate measured data; striped flows indicate calculated values. The mass of ‘oxides’ in the effluent flow, formed from fugitive oxygen, includes only the carbon and hydrogen components. See Methods for detailed calculations. Source data

Figure 4 gives a detailed breakdown of the mass conversion taking place inside the single-pass and multi-pass reactors. The inputs on the left of Fig. 4a,b represent the inputs shown on the left of Fig. 2d,e, respectively (recycled gases are not included). By recycling unreacted gases in the multi-pass reactor, throughput is reduced from 16.38 g h^−1^ to 1.15 g h^−1^, yet the CNT production is increased from 0.13 g h^−1^ to 0.17 g h^−1^.

The waste produced in the multi-pass process (0.88 g h^−1^) is lower than that in the single-pass process (16.25 g h^−1^). The single-pass waste stream shown in Fig. 4a comprises mostly hydrogen, but also contains >70% of the methane supply, which leaves unreacted, along with a further 10% of the carbon input in the form of hydrocarbon pyrolysis products (C_*x*_H_*y*_). By recycling these gases, the multi-pass reactor needs only enough methane input to ‘top up’ the carbon that is consumed in each pass of the furnace. The multi-pass process thus removes the need for an exogenous H_2_ input and enables a 10-fold reduction in the methane input and a 50% reduction in thiophene input (Fig. 4). The multi-pass process increases the amount of product from 0.13 g h^−1^ to 0.27 g h^−1^ (140 g h^−1^ m^−3^ to 293 g h^−1^ m^−3^), largely owing to the additional H_2_ product stream. The solid carbon loss is reduced twofold in the multi-pass process, from 1.1 g h^−1^ to 0.57 g h^−1^, but 71% of the carbon is still lost.

### Single-pass and multi-pass performance

**Fig. 5:**
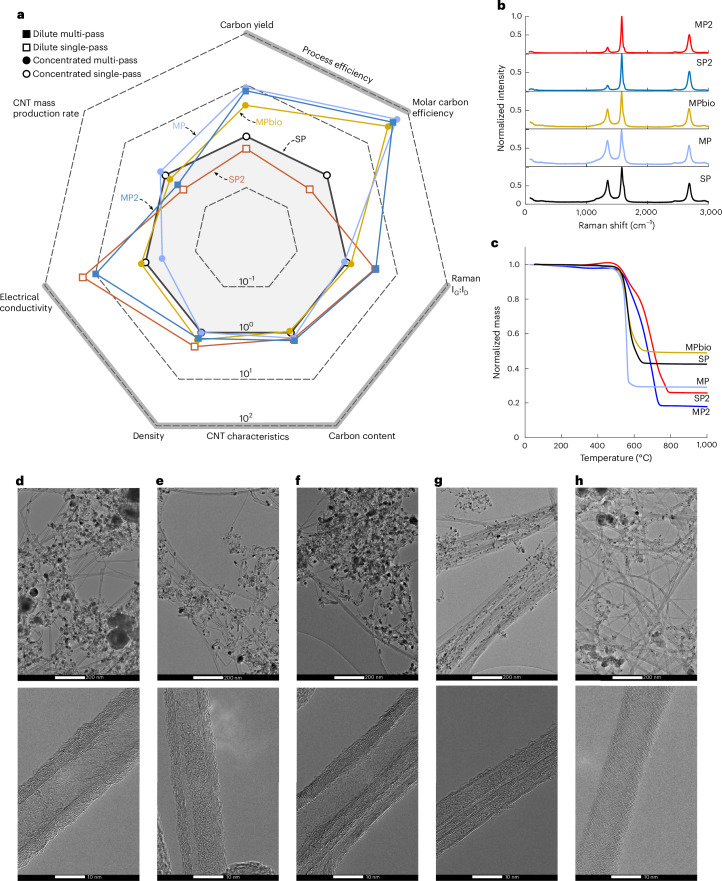
Comparison of single-pass and multi-pass process performance and CNT product. The results for five operating conditions, namely, concentrated single-pass (SP), concentrated multi-pass (MP), concentrated multi-pass with biogas (MPbio), dilute single-pass (SP2) and dilute multi-pass (MP2), are shown. **a**, Comparison of process performance metrics and material characteristics, plotted on a logarithmic scale normalized by the performance of the SP process. **b**,**c**, Normalized Raman spectra (**b**) and TGA curves (**c**). **d**–**h**, TEM images of CNT material produced with SP (**d**), MP (**e**), MPbio (**f**), SP2 (**g**) and MP2 (**h**). Source data

Both single-pass and multi-pass reactors were run with concentrated (SP and MP) and dilute (SP2 and MP2) precursor mixtures. An additional mixture of concentrated precursors with an impurity of 33 vol% CO_2_ in the methane (MPbio) was also tested to simulate unrefined bioderived methane. Supplementary Table 1 provides details of these reaction mixtures, and Fig. 5 and Table 1 summarize the results of these experiments (see Methods for details of the calculations). The performance of each process relative to the concentrated single-pass (SP) process is compared in Fig. 5a. Moving from the concentrated to the dilute single-pass (SP2) process there is a 40–70% decrease in CNT mass production, carbon yield and molar efficiency as less conversion takes place with fewer precursors inside the reactor. At the same time, some CNT properties increase in the dilute process, most notably the ratio of G-band to D-band measured with Raman spectroscopy (*I*_G_/*I*_D_) (3.8-fold) and electrical conductivity (17-fold). The need to balance throughput with CNT properties is reported in the literature and seems to be a caveat of FCCVD reactors^34,35^.

**Table 1:** Performance data and CNT characteristics of the five lab-scale processes (SP, MP, MPbio, SP2 and MP2), and the pilot-scale single-pass and modelled pilot-scale multi-pass processes

	SP	MP	MPbio	SP2	MP2	Pilot SP	Pilot MP
CNT mass production (g h^−1^)	0.132	0.165	0.0995	0.046	0.077	30.0	30.0
Carbon content (%)	64.41	75.53	58.89	78.33	85.37	95.02	95.02
Iron content (%)	29.66	20.39	34.26	18.06	12.19	4.15	4.15
Raman *I*_G_/*I*_D_	1.54	1.41	1.79	5.87	6.04	3.58	3.58
Electrical conductivity (S m^−1^)	3,643	1,593	4,165	63,669	35,470	–	–
Specific conductivity (S m^2^ kg^−1^)	26.74	11.48	21.87	233.2	213.9	–	–
Density (kg m^−3^)	137.9	139.1	190.4	273.0	166.1	–	–
Methane conversion (%)	27.20	91.90	95.44	–	–	85.0	95.0
Hydrogen production efficiency (%)	0	54.15	32.99	0	–	0	87.64
Carbon yield (%)	2.38	20.76	9.61	1.37	18.24	60.0	79.14
Molar process efficiency (%)	0.100	44.69	27.20	0.037	–	1.48	85.18
Molar carbon efficiency (%)	0.100	5.36	3.23	0.037	4.21	1.48	79.22
Mass process efficiency (%)	0.806	23.17	7.95	0.34	15.98	13.60	75.00
Volumetric productivity (kg m^−3^ h^−1^)	0.147	0.296	0.179	0	–	3.333	4.384
CNT volumetric productivity (kg m^−3^ h^−1^)	0.147	0.183	0.111	0.051	0.09	3.333	3.333
H_2_ volumetric productivity (kg m^−^^3^ h^−1^)	0	0.113	0.069	0	–	0	1.051

– indicates that no data points were obtained in this study.

Both the concentrated and dilute multi-pass processes (MP and MP2) show clear efficiency improvements in Fig. 5a, with a 7.7–8.7-fold increase in carbon yield and 42–53-fold increase in molar carbon efficiency compared with the concentrated single-pass process. While the dilute single-pass process shows a drop in efficiency compared with the concentrated single-pass, the dilute multi-pass still proffers an efficiency improvement, although its CNT production rate decreases by 42%. When the additional hydrogen product stream from the concentrated multi-pass process is considered, its overall molar process efficiency increases to 44.69%, 446-fold higher than the concentrated single-pass process, which operates at 0.1% molar efficiency, typical for lab-scale FCCVD reactors^20^. Moving from the single-pass to the multi-pass process proffers an increase in CNT mass production rate of 25% in the concentrated case and of 67% in the dilute case.

The introduction of CO_2_ impurities into the multi-pass process (MPbio) reduces its efficiency compared with running on pure methane. However, it still exhibits a 4- and 32-fold increase in carbon yield and molar carbon efficiency compared with the single-pass process, respectively, but suffers a 25% reduction in CNT mass production. The reduced efficiency and productivity of the MPbio process compared with the MP process is attributed to CO_2_ oxidizing carbon to form CO, which acts as a detrimental carbon sink (see Supplementary Note 3 for more details and Supplementary Fig. 5 for oxide concentrations in the process gas).

Comparing the Raman spectra presented in Fig. 5b, the materials produced with the concentrated reaction mixtures show a range of *I*_G_/*I*_D_ ratios (1.4–1.8), while those produced with the dilute mixtures show very similar *I*_G_/*I*_D_ ratios of ~6. The thermogravimetric analysis (TGA) curves in Fig. 5c show that the multi-pass processes produce material with less iron impurity than their single-pass counterparts, as evidenced by the ~30% smaller residual mass. Conversely, the MPbio material contains 15% more iron than the single-pass material. The materials produced with the dilute recipes (SP2 and MP2) show more mass loss at high temperature (>600 °C), indicating the presence of CNTs with more graphetization and fewer defects^23^. The materials produced under dilute conditions are more electrically conductive (>10-fold) than the materials from concentrated mixtures and also more dense. The dilute single-pass material exhibits the highest electrical conductivity (63,669 S m^−1^), density (273 kg m^−3^) and specific conductivity (233.2 S m^2^ kg^−1^).

Structural differences in the CNT materials are visible in their transmission electron microscopy (TEM) images, as shown in Fig. 5d–h. Moving from the concentrated (Fig. 5d–f) to the dilute recipes (Fig. 5g,h), the CNTs appear longer, forming more coherent networks. In particular, the dilute single-pass material (Fig. 5g) shows dense bundles with diameters of ~100 nm, containing many CNTs, possibly explaining the high density and electrical conductivity of this material. Comparing the concentrated multi-pass material in Fig. 5e with the single-pass material in Fig. 5d, there is a visible reduction in the number and size of iron nanoparticles, corroborating the lower residual mass revealed by TGA. The CNTs in the concentrated multi-pass and MPbio materials appear shorter than the CNTs in the single-pass material, and all three samples are composed of multi-wall CNTs with a diameter of ~10 nm. The CNTs produced with the dilute recipes (SP2 and MP2) are also multi-walled, albeit with fewer walls. Many of the CNTs in the MPbio material (Fig. 5f) exhibit ‘herringbone’ walls, where the graphite planes are diagonal to the axis of the CNTs, possibly a result of the additional CO_2_ impurity.

### Mass conversion in the pilot-scale process

**Fig. 6:**
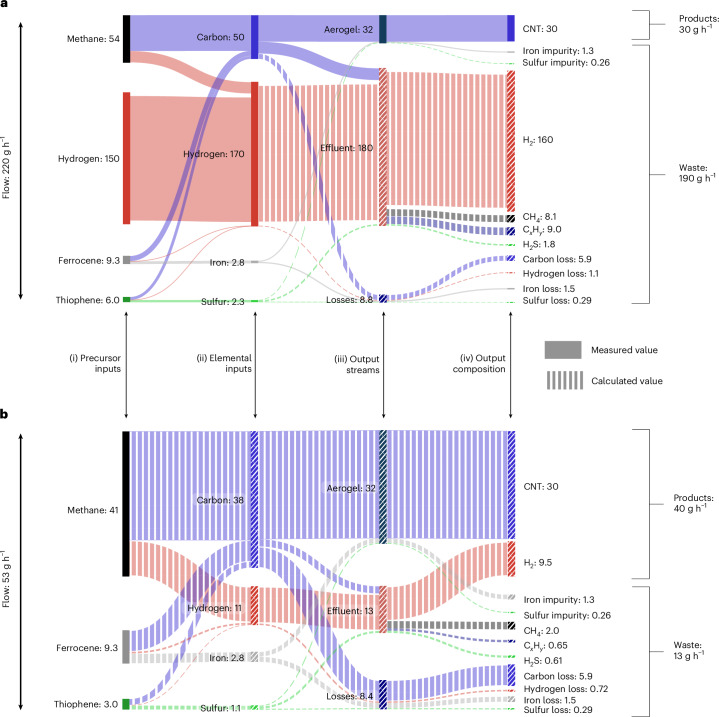
Mass conversion in the pilot-scale processes. **a**,**b**, Mass conversion (g h^−1^) in pilot-scale single-pass (**a**) and modelled pilot-scale multi-pass (**b**) processes, with the multi-pass process demonstrating much more efficient precursor conversion. The Sankey diagrams show the conversion of mass from precursor inputs (i) and elemental inputs (ii) to output streams (iii), as well as a breakdown of the chemical composition of the output streams (iv). Solid flows indicate measured data; striped flows indicate calculated values. See Methods for more details on the construction of these diagrams. Source data

To assess the scale-up implications of the multi-pass process, we collected data from a pilot-scale single-pass reactor operated by Tortech Nano Fibers to construct the Sankey diagram presented in Fig. 6a. The pilot reactor operates at a higher CNT production rate (30 g h^−1^), volumetric productivity (3.4 kg m^−3^ h^−1^) and carbon yield (60%) than the lab-scale reactor. The solid carbon loss is 5.9 g h^−1^, one-fifth of the size of the CNT product stream. However, the pilot single-pass process in Fig. 6a is dominated by the large hydrogen waste stream, with a net H_2_ loss during the process.

Applying the reaction efficiencies of the multi-pass process to a pilot-scale reactor indicates that a pilot-scale multi-pass process could achieve efficient conversion of methane into CNTs and hydrogen. Figure 6b shows the mass conversion for a pilot-scale reactor running in the multi-pass configuration, producing 30 g h^−1^ of CNTs. The reduction in waste combined with the increase in product make the multi-pass process 57-fold more efficient than the single-pass process on a molar basis. The dominant carbon loss stream exhibited by the lab-scale multi-pass process in Fig. 4b is reduced at the pilot scale, becoming secondary to the CNT and hydrogen product streams in Fig. 6b. Overall, the process outputs 75% product by mass, with the process producing CNTs and hydrogen in a 3:1 mass ratio at a rate of 4.4 kg m^−3^ h^−1^, with an 88% hydrogen production efficiency. While this is a notable improvement in efficiency compared with the pilot single-pass process, the pilot multi-pass process still outputs 25% waste by mass, meaning that further improvements should be pursued on scaling from pilot to industrial reactors.

### Multi-pass FCCVD compared with other pyrolysis technologies

**Fig. 7:**
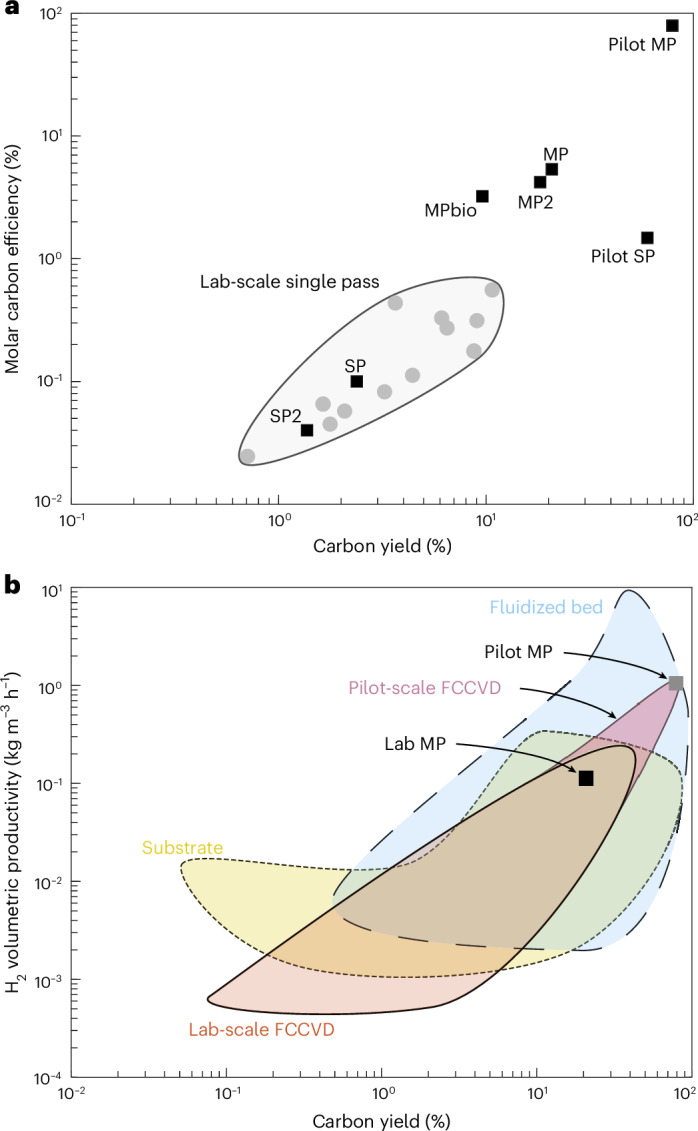
Comparison of the multi-pass and single-pass process performance with literature data. **a**, CNT production efficiency in terms of molar carbon efficiency and carbon yield compared with data for lab-scale FCCVD reactors presented by Weller at al.^20^. **b**, H_2_ productivity and carbon mass conversion compared with data presented by Glerum and Boies^26^ for FCCVD, substrate and fluidized bed CNT reactors. Source data

The single-pass processes explored in this study exhibit relatively low molar carbon efficiency (0.04–0.1%) and carbon yield (1.4–2.4%), whereas the multi-pass processes are able to achieve a much higher molar carbon efficiency (4.2–5.4%) and carbon yield (18.2–20.8%), as shown in Fig. 7a. Scaling up the reactor for the pilot single-pass process achieves a carbon yield of 60%, much higher than the lab-scale processes. The pilot multi-pass process model predicts a carbon yield and molar carbon efficiency of 79%, along with a hydrogen volumetric productivity of 1.1 kg m^−3^ h^−1^, approaching the efficiencies of fluidized bed systems^26^, as shown in Fig. 7b. The multi-pass reactor is compared with other ‘hydrogen-first’ pyrolysis reactors in Supplementary Fig. 6.

## Discussion

This study has demonstrated that an FCCVD CNT reactor can operate in a multi-pass configuration with ~99% recycled process gas, requiring no exogenous hydrogen supply. The multi-pass reactor produced H_2_ with 84.7 vol% purity and CNT aerogel, both were continuously extracted from the reactor. Compared with a conventional single-pass FCCVD reactor, the multi-pass reactor demonstrated an 8.7-fold improvement in carbon yield and 446-fold improvement in molar process efficiency. These efficiency gains are accompanied by comparatively small changes to the characteristics of the CNT aerogel. The dilute multi-pass recipe produced CNTs with a Raman *I*_G_/*I*_D_ value of 6, comparable to the CNTs used by Zhang et al.^16^ to create a fibre with a tensile strength of 8 GPa. Applying these findings to data collected from a commercial single-pass CNT reactor, we calculated that a pilot-scale multi-pass reactor could produce CNTs and hydrogen in a 3:1 mass ratio, with 75% of the mass throughput converted into useful products.

The co-production of CNTs and hydrogen has previously been reported using fluidized bed reactors^21–24^. These processes require periodic removal of CNTs and regeneration of the catalyst, so the FCCVD reactor’s ability to continuously extract CNTs and synthesize fresh catalyst is advantageous. However, fluidized bed reactors use more concentrated methane feedstock (25–100 vol% CH_4_) than FCCVD reactors (5–10 vol% CH_4_), allowing greater hydrogen productivity. Fluidized bed reactors are characteristic of ‘hydrogen-first’ processes such as molten metal, thermal pyrolysis and plasma pyrolysis reactors^10,17,18,36^. These reactors enable the high-production synthesis of turquoise hydrogen from concentrated methane feedstocks, while producing relatively low-value solid carbon. The fluidized bed reactors typically produce CNTs of large diameter (>50 nm) and low *I*_G_/*I*_D_ ratio (~1), and are collected in powder form. Recent innovations in rotary kiln reactors enable the continuous synthesis of CNTs, potentially with smaller diameters than those from fluidized bed reactors, but the CNTs are still harvested as powder^25^.

The markets for carbon powders are unlikely to accommodate the volumes produced while satisfying the global hydrogen demand. For example, producing turquoise hydrogen to meet today’s hydrogen demand (~100 MtH_2_ yr^−1^) would result in the production of ~300 Mt yr^−1^ of solid carbon, that is, >10-fold larger than the current carbon black market (~18 Mt yr^−1^)^26,27^. The production of primarily powdered CNTs is being scaled up to serve the battery electrode market, but is currently only ~20 kt yr^−1^ (ref. ^37^). As a ‘carbon-first’ approach, the multi-pass reactor produces CNTs with a small diameter (~10 nm) and high *I*_G_/*I*_D_ ratio (up to 6) that naturally form bulk materials within the reactor and can be densified for further property enhancement^11,16,38^. Using bulk CNT materials as replacements for materials such as steel, aluminium and copper present the opportunity to not only increase CNT use but to realize additional CO_2_ emissions reductions by displacing CO_2_-intensive materials. Furthermore, the fact that multi-pass FCCVD can use biomass-derived methane offers a means for the net sequestration of carbon from the atmosphere within useful materials.

Huntsman Corporation announced long-term goals to scale up their FCCVD CNT process to 1 MtCNT yr^−1^ (ref. ^39^). Assuming CNT and hydrogen production in the same proportion as the pilot multi-pass process presented here, this plant would produce 330 ktH_2_ yr^−1^. Three hundred such plants could supply today’s H_2_ demand (~100 MtH_2_ yr^−1^) and consume ~15% of global natural gas (see Supplementary Note 4 for further details).

The global capacity of FCCVD methane pyrolysis systems is currently small, although precise numbers are unknown. Deploying this technology at scale presents a number of challenges. The first is the solid loss generated by the reactor. The pilot-scale reactor reduces the proportion of loss relative to the lab-scale reactor, but 15% of carbon is still lost. If such reactors are to process megatonnes of methane, the proportion of loss must be reduced by orders of magnitude. Similarly, the pilot multi-pass reactor consumes ferrocene and thiophene at 25% and 7.5% the rate of methane, respectively. Working at the megatonne scale, this may become prohibitive and will demand innovation in terms of catalyst delivery and use. There is scope for large improvements in catalyst efficiency in FCCVD reactors as it has been estimated that <0.1% of catalyst particles grow CNTs^34^. In addition, switching to elemental catalyst precursors may reduce costs.

A broader challenge is the ~3% leakage of methane from natural gas supplies^40^, contributing ~18% of total methane flux to the atmosphere^41^. Hydrogen that leaks into the atmosphere can also cause global warming^42^, so any wide-scale deployment of methane pyrolysis must address both upstream methane and downstream hydrogen leakage. Conversely, the FCCVD process allows biogas conversion to functional carbons, enabling the net removal of atmospheric carbon into functional materials^43^. In addition, the diversion of natural gas away from combustion applications and into pyrolysis processes could reduce CO_2_-equivalent emissions from existing infrastructure; without intervention, these emissions alone will exceed the budget for 1.5 °C of global warming^44^. Overall, the multi-pass FCCVD process for the co-production of bulk CNT materials and turquoise hydrogen could reduce greenhouse emissions, but considerable development is needed to deploy this technology on a meaningful scale.

## Methods

### Precursors

N4.5 (zero-grade) argon, methane and CO_2_, and N5.0 (CP-grade) hydrogen and nitrogen were supplied by BOC. Ferrocene powder (98% purity) and liquid thiophene (>99% purity) were supplied by Sigma-Aldrich. The MPbio precursor contained 67 vol% CH_4_ and 33 vol% CO_2_, representative of unrefined landfill and biogas (47–70 vol% methane and 32–43 vol% CO_2_)^28^.

### Dispensers

Gas flows were controlled by Alicat MC-Series MFCs with the following maximum flow rates and calibration gases: process gas (5 standard l min^−1^, H_2_), ferrocene carrier gas (1 standard l min^−1^, H_2_), thiophene carrier gas (500 standard cm^3^ min^−1^, H_2_), methane (500 standard cm^3^ min^−1^, CH_4_), recycled process gas (2 standard l min^−1^, H_2_), FTIR nitrogen purge (5 standard l min^−1^, N_2_) and CO_2_ (1 standard l min^−1^, CO_2_). Standard temperature and pressure were taken to be 273 K and 1 bar, respectively.

Ferrocene was dispensed by flowing hydrogen carrier gas through a sublimation pack held at constant temperature, allowing dispensation rate calculations using the vapour pressure data from Fulem et al.^45^. Tubes carrying ferrocene vapour from the sublimation pack to the furnace were heated to 160 °C to prevent ferrocene condensing inside the pipework. Thiophene was dispensed by bubbling hydrogen through liquid thiophene cooled to 0.5 °C in an ice bath; the dispensation rate was calculated using the Antoine equation provided by NIST^46^. Fully saturated carrier gas was assumed for both precursors.

### Reactor

CNTs were synthesized using the FCCVD process first reported by Li et al.^19^. A collection chamber was fabricated by Mackworks Precision Engineering to allow continuous winding of the CNT aerogel onto a rotating roller. Figure 3 shows a schematic of the reactor used in this study and Supplementary Fig. 4 shows a photograph of the reactor. The reactor could run in the single-pass configuration with the recycle MFC closed and the SP/MP selector valve taking hydrogen from the exogenous gas supply. In the single-pass configuration, all process gas exits the collection chamber through the water trap and bubbler.

Alternatively, the reactor could run in the multi-pass configuration by closing the exogenous hydrogen supply and switching the SP/MP selector valve to accept recycled process gas. In the multi-pass configuration, process gas from the collection chamber is pumped through the recycle MFC and back to the injector via the various MFCs and catalyst dispensers. During multi-pass operation, additional gas produced by the pyrolysis reaction left the reactor through the exhaust line. The exhaust line was fitted with a 2.3 kPa check valve to ensure that air could not back-flow into the reactor. Precursor recipes are provided in Supplementary Table 1, along with an explanation of the differences between the single-pass and multi-pass recipes in Supplementary Note 5.

The reaction took place inside an alumina tube of 580 mm length × 45 mm inner diameter × 50 mm outer diameter. The reaction tube was heated inside a Carbolite Gero tube furnace with a set point of 1,300–1,350 °C. Gases and precursors were injected through a stainless steel tube of 3 mm inner diameter × 6.35 mm outer diameter that protrudes 80 mm into the reaction tube. Gases and products exited the reaction tube into a stainless steel collection chamber where the CNT aerogel was wound onto an aluminium roller of 50 mm diameter rotating at ~60 r.p.m. A single CNT mat was collected for each experiment, made by winding many layers of aerogel on top of each other on the roller. A 500 mm × 6.35 mm stainless steel rod could be inserted through an Ultra-Torr fitting in the collection chamber and into the end of the reaction tube; this allowed CNT aerogel to be ‘fished’ from the reactor at the start of operation and in case winding was interrupted during an experiment.

Between experiments, the reactor was cooled to ambient temperature and flushed with argon to remove flammable gases and then with air to make it safe to open and clean. CNT samples were removed from the collection chamber, and the reaction tube and collection chamber were cleaned of soot and other solid residues using acetone and isopropyl alcohol. Once clean, the reactor was sealed and heated under a flow of air. Supplementary Note 6 describes the start-up and shut-down procedure of the reactor in detail.

### Measurement of CNT production

Carbon nanotubes were collected from the reactor after each experiment in the form of a mat collected on the roller and material collected on the stainless steel ‘fishing rod’. Both samples were weighed using a microbalance (A&D BM-252). The CNT mass production for a given experiment was taken to be the sum of the mass of these two samples. The CNT mass production rate was calculated by dividing the CNT mass production by the length of the experiment, measured to the nearest minute using a Lenovo P50 laptop.

### CNT characterization

Samples were cut from the CNT mats for characterization. Raman spectroscopy was carried out using a Horiba XploRA PLUS Raman microscope with a 532-nm laser, 10% power, 450–850 nm grating and ×50 objective. TGA was carried out using a Mettler Toledo TGA/DSC 2 instrument under a flow of air of 25 standard cm^3^ min^−1^. The samples were heated from 25 °C to 1,000 °C at a rate of 5 °C min^−1^. Electrical conductivity measurements were performed on a bespoke four-point probe jig connected to a milliohm meter (Aim-TTi BS407) to measure the resistance between three locations along the length of 100 mm × 10 mm strips of mat. The thickness of these strips was determined by averaging micrometre measurements taken at three positions along their length. Their mass was also measured using a microbalance, allowing their density to be calculated. TEM samples were prepared by dispersing CNT samples in ethanol by sonication for ~1 h, until the ethanol was visibly slightly darker owing to the dispersion of CNTs. The CNTs were then drop cast onto copper TEM grids with holey carbon support films. TEM analysis was carried out using a Thermo Fisher Scientific Talos F200X G2 TEM microscope.

### Gas measurements

During multi-pass operation, the effluent flow rate was measured with an Alicat MW-Series low-pressure-drop MFM with a full-scale flow rate of 1,000 standard cm^3^ min^−1^, shown in the upper right of Fig. 3. Mass flow rate measurements were corrected for gas composition according to Supplementary Note 7. During single-pass operation, the MFM was replaced by a Sensidyne Gilibrator 2 device with a 6 l min^−1^ standard flow cell. The hydrogen concentration in the effluent flow was measured using a V&F HSense mass spectrometer with an accuracy equal to 3% of the measured value. The HSense was located downstream of the effluent MFM, shown in the top right of Fig. 3. FTIR was conducted using a Bruker Matrix MG5 FTIR spectrometer with a path length of 5 m (0.5 cm^−1^ spectral resolution) tracking CH_4_, C_2_H_6_, C_2_H_4_, C_2_H_2_, C_6_H_6_, H_2_O, CO_2_ and CO. During multi-pass operation, the FTIR spectrometer was located downstream of the recycle MFC (Fig. 3). Recycled process gas was analysed in the FTIR spectrometer and then returned to the reactor in a closed loop. The larger recycle flow provided a shorter response time (~20 s) than the smaller effluent flow, and the effluent and recycled process gases had the same composition. To characterize the single-pass exhaust gases, the FTIR spectrometer was positioned on the exhaust line.

### Iron and sulfur content

Iron content was calculated from the TGA residual mass by assuming that the residual mass was Fe_2_O_3_, such that 69.94% of the residual mass would be the iron from the aerogel and the remaining 30.06% would be oxygen gained during the TGA experiment. Catalyst particle sulfur content was estimated to be 20 wt% based on the model of active catalyst particle chemistry presented by Weller et al.^20^. We acknowledge that the specific role of sulfur in the process, and its concentration in catalyst particles, is not completely understood at the time of writing and that this is an estimate.

### Loss quantities

Losses were calculated as the total mass of each element injected into the process minus the amount of that element measured leaving the reactor in the aerogel and effluent gas stream. This indicates the total mass that cannot be measured leaving the reactor. We made the assumption that any mass not leaving the reactor accumulates inside the reactor as loss, for example, on the walls of the reactor and in particle filters. This allows a quantitative analysis of the performance of the reactor with respect to mass conversion into useful product and the mass converted into waste products or loss. However, it does not confirm the precise chemistry, mechanisms or location of loss inside the reactor.

Iron in the reactor forms solid particles that are removed from the effluent and recycled gas flows by particle filters. Therefore, all iron not accounted for in the aerogel must take the form of solid loss. The mass of sulfur loss was assumed to equal 20% of the mass of iron loss (assuming the same nanoparticle chemistry as the catalyst in the aerogel). Hydrogen loss was assumed to accumulate in the form of hydrocarbon molecules within the amorphous carbon loss, thus the ratio of carbon loss to hydrogen loss is dependent on the chemistry of this amorphous carbon.

### Sankey diagrams

The Sankey diagrams in Fig. 4 collate the inputs for each process and the data collected on the outputs, and unmeasured values were calculated. The precursor recipes and input rates are provided in Supplementary Table 1. The input rates of elemental carbon, hydrogen, sulfur and iron were then calculated from these data by summing the contribution of each element from each of the precursors. The mass production rate of the aerogel, effluent gas and loss streams were calculated as described in the relevant sections above. Effluent H_2_S was calculated as the mass of sulfur not accounted for in the aerogel and solid loss. Oxides were detected in the exhaust of the lab-scale process, primarily as CO and H_2_O. The source of the oxygen is believed to be leaks in the reactor’s pipework, impurities in the feed gases, etching from the alumina (aluminium oxide) reaction tube or some combination thereof. To simplify the Sankey diagrams, the fugitive oxygen was not included and the mass of ‘oxides’ in the effluent flow accounts only for the mass of carbon and hydrogen bound in the oxide species (see Supplementary Note 4 for a more detailed analysis of oxides in the effluent streams).

The Sankey diagram representing the pilot-scale single-pass process in Fig. 6a was constructed using data collected by ourselves at the Tortech pilot-scale plant in Ma’alot Tarshiha, Israel, and by characterizing the material produced by this process (Table 1) and from the recipes and performance data provided by Tortech Nano Fibres. Supplementary Table 2 contains the mass flow rates of the precursors provided by Tortech, enabling the calculation of the elemental input rates. The production rate of CNTs was 30 g h^−1^ for this recipe. The iron impurity in the aerogel was measured by TGA and the sulfur impurity was calculated at 20 wt% of the iron impurity, as above. The loss streams and effluent H_2_S were also calculated as above. The mass flow of effluent methane was known from the methane conversion efficiency (85%); however, the concentrations of the other effluent gas species were not measured. The effluent concentrations of C_*x*_H_*y*_ were assumed to be the same as in the lab-scale single-pass process, while oxide species were ignored. The ratio of hydrogen loss/solid carbon loss was assumed to match the lab-scale single-pass process, and any remaining hydrogen was assumed to leave the reactor in the effluent stream. The mass of effluent hydrogen not accounted for in species such as H_2_S, CH_4_ and C_*x*_H_*y*_ was assumed to be in the form of H_2_, yielding the mass flow rate of effluent H_2_ and defining the effluent flow rate. The mass of carbon loss was then iterated until the total mass of carbon in the aerogel, effluent and loss stream matched the carbon input, at which point all mass inputs and outputs of the reactor were balanced.

The effluent and loss flows shown with stripes in Fig. 6a were calculated rather than measured, thus the relative distribution of loss between the solid and gas phase was based on calculation. However, the precursor flow rates and CNT production rates in Fig. 6a are measured values, and thus the sizes of the product and waste streams are based on measurements. The process efficiencies and carbon yields are based on the size of these two streams and are thus based solely on measured values.

The Sankey diagram in Fig. 6b was constructed by combining the data collected for the pilot-scale single-pass process in Fig. 6a and the performance improvements demonstrated by the lab-scale multi-pass process. The performance improvements for the pilot scale cannot be as large as those seen in the lab because the pilot-scale single-pass process is already much more efficient (Table 1). The pilot-scale multi-pass process was designed to produce 30 g h^−1^ CNTs like the pilot-scale single-pass process, with the same quantities of iron and sulfur impurity. The exogenous hydrogen supply was completely removed assuming that process gas will circulate in a closed loop as in the lab-scale multi-pass process. The flow rate of ferrocene was kept the same, while the thiophene flow was halved, in line with the lab-scale multi-pass process. A methane conversion efficiency of 95% was applied to the pilot-scale multi-pass process based on the 92% exhibited by the lab-scale process. Effluent methane concentration was calculated on the basis of this conversion efficiency. Effluent pyrolysis products were assumed to have the same concentrations as in the lab-scale multi-pass process, and effluent oxides were ignored. Carbon loss was assumed to remain the same in the pilot-scale single-pass and multi-pass reactors, and the ratio of carbon loss/hydrogen loss was assumed to be same as in the lab-scale multi-pass reactor. The mass of iron loss was calculated as the mass of iron not accounted for in the aerogel, and sulfur loss was calculated as 20% of the iron loss. The mass of effluent hydrogen and sulfur was calculated as the mass not accounted for in the aerogel and loss streams, providing the mass flow rate of effluent H_2_S. Effluent H_2_ was calculated as the mass of effluent hydrogen not accounted for in other effluent species, defining the effluent flow rate. The methane input into the process was then iterated until the inflows and outflows of carbon and hydrogen were balanced.

### Efficiencies

CH_4_ conversion is defined as

2 $${\rm{C{H}}}_{4}\,\mathrm{conversion}\,=\frac{\dot{m}({\rm{C{H}}}_{4},\,\mathrm{injector})\,-\,\dot{m}({\rm{C{H}}}_{4},\,\mathrm{exhaust})}{\dot{m}({\rm{C{H}}}_{4},\,\mathrm{injector})}$$

measured during steady-state operation, where $\dot{m}({\rm{C{H}}}_{4},\,\mathrm{injector})$ and $\dot{m}({\rm{C{H}}}_{4},\,\mathrm{exhaust})$ are the mass flow rates of CH_4_ in the injector and exhaust, respectively, where *ṁ* is used to represent a mass flow rate. CH_4_ conversion describes the amount of methane converted into different species inside the reactor.

Hydrogen production efficiency in a multi-pass FCCVD reactor must consider the hydrogen liberated from ferrocene and thiophene, along with the primary contribution from methane. H_2_ production efficiency is thus defined as

3 $${{\rm{H}}}_{2}\,\text{production efficiency}\,=\,\frac{\dot{m}({{\rm{H}}}_{2},\,\mathrm{exhaust})}{\dot{m}({\rm{H}},\,\mathrm{injector})}$$

where $\dot{m}({{\rm{H}}}_{2},\,\mathrm{exhaust})$ is the mass flow rate of H_2_ leaving the reactor and $\dot{m}({\rm{H}},\,\mathrm{injector})$ is the mass flow rate of hydrogen injected into the reactor during steady-state operation.

Carbon yield is defined as

4 $$\,\text{Carbon yield}\,=\,\frac{\text{Mass of carbon in aerogel (g)}}{\text{Total mass of carbon injected (g)}}$$

during steady-state operation. It describes the fraction of the total carbon injected into the system that is converted into useful carbon products.

Molar process efficiency is defined as

5 $$\text{Molar process efficiency}\,=\,\frac{\text{Amount of useful product (mol)}}{\text{Total precursor input (mol)}}$$

measured during steady-state operation. Note that the amounts of product and precursors are measured in terms of the number of moles of atoms, rather than the number of moles of molecules, such that molar efficiency describes the number of moles of atoms that are converted into useful products. In the single-pass process, useful product is limited to the carbon aerogel. In the multi-pass process, useful product includes both the aerogel and the effluent hydrogen gas. Molar carbon efficiency is calculated in the same way as the molar process efficiency, but considers only the moles of carbon product:

6 $$\text{Molar carbon efficiency}\,=\,\frac{\text{Amount of carbon product (mol)}}{\text{Total precursor input (mol)}}.$$

Mass-based process efficiency is defined similarly to molar process efficiency, but measures the mass of precursors and products rather than the number of moles:

7 $$\text{Mass-based process efficiency}\,=\,\frac{\text{Mass of useful product (g)}}{\text{Total precursor input (g)}}.$$

## Supplementary information

Supplementary Information Supplementary Tables 1 and 2, Supplementary Figs. 1–6, Supplementary Notes 1–7 and Supplementary References.

Supplementary Video 1 Aaerogel being extracted from the reactor and wound onto the roller. It then shows hydrogen gas leaving the reactor through a bubbler.

Supplementary Data 1 Source data for Supplementary Figs. 1–3 and 5.

## Source data

Source Data Fig. 1 Unprocessed images used in the ‘metre’, ‘micrometre’, ‘nanometre’ and renders of the reactor used in Fig. 1a.

Source Data Fig. 2 Source_Data_Figure_2_Images.pdf contains unprocessed images of the aerogel and mat used in Fig. 2b,c, and unprocessed render of the reactor used in Fig. 2a. Source_Data_Figure_2.xlsx contains the H_2_ concentrations and effluent flows used to calculate the average H_2_ concentration and effluent flow in Fig. 2a; data used to calculate the product, waste and recycle stream proportions in Fig. 2d,e.

Source Data Fig. 4 Data and calculations used to produce the Sankey diagrams.

Source Data Fig. 5 Source_Data_Figure_5.xlsx contains the data for each repeat used to obtain the average values presented in. Fig. 5a. Source_Data_Figure_5_images.pdf contains the unprocessed TEM images used in Fig. 5d–h.

Source Data Fig. 6 Data and calculations used to produce the Sankey diagrams.

Source Data Fig. 7 Plotted data points from this work.

## Data Availability

All of the data supporting the findings of this study are available within the paper and its Supplementary Information. Source data are provided with this paper.

## References

[1] Schemme, S. et al. H_2-_based synthetic fuels: a techno-economic comparison of alcohol, ether and hydrocarbon production. *Int. J. Hydrogen Energy* **45**, 5395–5414 (2020).

[2] Madadi Avargani, V., Zendehboudi, S., Cata Saady, N. M. & Dusseault, M. B. A comprehensive review on hydrogen production and utilization in North America: prospects and challenges. *Energy Convers. Manage.* **269**, 115927 (2022).

[3] Henry, A., Prasher, R. & Majumdar, A. Five thermal energy grand challenges for decarbonization. *Nat. Energy* **5**, 635–637 (2020).

[4] Global hydrogen demand in the Net Zero Scenario, 2022-2050. *IEA* https://www.iea.org/data-and-statistics/charts/global-hydrogen-demand-in-the-net-zero-scenario-2022-2050 (2023).

[5] Diab, J., Fulcheri, L., Hessel, V., Rohani, V. & Frenklach, M. Why turquoise hydrogen will be a game changer for the energy transition. *Int. J. Hydrogen Energy* **47**, 25831–25848 (2022).

[6] Ritchie, H. & Roser, M. Sector by sector: where do global greenhouse gas emissions come from? *Our World in Data* https://ourworldindata.org/ghg-emissions-by-sector (2024).

[7] Rahimi, N. et al. Solid carbon production and recovery from high temperature methane pyrolysis in bubble columns containing molten metals and molten salts. *Carbon* **151**, 181–191 (2019).

[8] Lamy, C. & Millet, P. A critical review on the definitions used to calculate the energy efficiency coefficients of water electrolysis cells working under near ambient temperature conditions. *J. Power Sources* **447**, 227350 (2020).

[9] Muradov, N. Hydrogen via methane decomposition: an application for decarbonization of fossil fuels. *Int. J. Hydrogen Energy* **26**, 1165–1175 (2001).

[10] Qian, J. X. et al. Methane decomposition to pure hydrogen and carbon nano materials: state-of-the-art and future perspectives. *Int. J. Hydrogen Energy* **45**, 15721–15743 (2020).

[11] Zhang, X. et al. Simultaneously enhanced tenacity, rupture work, and thermal conductivity of carbon nanotubes fibers by increasing the effective tube contribution. *Sci. Adv.* **8**, eabq3515 (2022). 36516257 10.1126/sciadv.abq3515 PMC9750159

[12] Behabtu, N. et al. Strong, light, multifunctional fibers of carbon nanotubes with ultrahigh conductivity. *Science* **339**, 182–186 (2013). 23307737 10.1126/science.1228061

[13] Gspann, T. S. et al. High thermal conductivities of carbon nanotube films and micro-fibres and their dependence on morphology. *Carbon* **114**, 160–168 (2017).

[14] Laurent, C., Flahaut, E. & Peigney, A. The weight and density of carbon nanotubes versus the number of walls and diameter. *Carbon* **48**, 2994–2996 (2010).

[15] Taylor, L. W. et al. Improved properties, increased production, and the path to broad adoption of carbon nanotube fibers. *Carbon* **171**, 689–694 (2021).

[16] Zhang, X. et al. Carbon nanotube fibers with dynamic strength up to 14 GPa. *Science* **384**, 1318–1323 (2024). 38900888 10.1126/science.adj1082

[17] Fan, Z., Weng, W., Zhou, J., Gu, D. & Xiao, W. Catalytic decomposition of methane to produce hydrogen: a review. *J. Energy Chem.* **58**, 415–430 (2021).

[18] Abánades, A. et al. Experimental analysis of direct thermal methane cracking. *Int. J. Hydrogen Energy* **36**, 12877–12886 (2011).

[19] Li, Y.-L., Kinloch, I. A. & Windle, A. H. Direct spinning of carbon nanotube fibers from chemical vapor deposition synthesis. *Science* **304**, 276–278 (2004). 15016960 10.1126/science.1094982

[20] Weller, L. et al. Mapping the parameter space for direct-spun carbon nanotube aerogels. *Carbon* **146**, 789–812 (2019).

[21] Sun, E. et al. A semi-continuous process for co-production of CO_2_-free hydrogen and carbon nanotubes via methane pyrolysis. *Cell Rep. Phys. Sci.* **4**, 101338 (2023).

[22] Parmar, K. R., Pant, K. K. & Roy, S. Blue hydrogen and carbon nanotube production via direct catalytic decomposition of methane in fluidized bed reactor: capture and extraction of carbon in the form of CNTs. *Energy Convers. Manage.* **232**, 113893 (2021).

[23] Kutteri, D. A., Wang, I.-W., Samanta, A., Li, L. & Hu, J. Methane decomposition to tip and base grown carbon nanotubes and CO_*x*_-free H_2_ over mono- and bimetallic 3d transition metal catalysts. *Catal. Sci. Technol.* **8**, 858–869 (2018).

[24] Wang, I.-W., Kutteri, D. A., Gao, B., Tian, H. & Hu, J. Methane pyrolysis for carbon nanotubes and CO_*x*_-free H_2_ over transition-metal catalysts. *Energy Fuels* **33**, 197–205 (2019).

[25] Silvy, R. A. P. & Arthur, D. J. System and method for synthesizing carbon nanotubes and hybrid materials via catalytic chemical deposition. US patent US20230109092A1 (2023).

[26] Glerum, M. W. J. & Boies, A. M. in *Advances in Chemical Engineering*. *Turquoise Hydrogen: An Effective Pathway to Decarbonization and Value Added Carbon Materials* Vol. 61 (eds Pelucchi, M. & Maestri, M.) 133–192 (Academic Press, 2023).

[27] Pasquali, M. & Mesters, C. We can use carbon to decarbonize—and get hydrogen for free. *Proc. Natl Acad. Sci. USA* **118**, e2112089118 (2021). 34321359 10.1073/pnas.2112089118 PMC8346849

[28] Rasi, S. *Biogas Composition and Upgrading to Biomethane*. PhD thesis, Univ. of Jyväskylä (2009).

[29] Guéret, C., Daroux, M. & Billaud, F. Methane pyrolysis: thermodynamics. *Chem. Eng. Sci.* **52**, 815–827 (1997).

[30] Hoecker, C., Smail, F., Bajada, M., Pick, M. & Boies, A. Catalyst nanoparticle growth dynamics and their influence on product morphology in a CVD process for continuous carbon nanotube synthesis. *Carbon* **96**, 116–124 (2016).

[31] Hoecker, C., Smail, F., Pick, M., Weller, L. & Boies, A. M. The dependence of CNT aerogel synthesis on sulfur-driven catalyst nucleation processes and a critical catalyst particle mass concentration. *Sci. Rep.* **7**, 14519 (2017). 29109427 10.1038/s41598-017-14775-1 PMC5673953

[32] Boies, A. M. et al. Agglomeration dynamics of 1D materials: gas-phase collision rates of nanotubes and nanorods. *Small* **15**, 1900520 (2019). 10.1002/smll.201900520 31120182

[33] Kateris, N., Kloza, P., Qiao, R., Elliott, J. A. & Boies, A. M. From collisions to bundles: an adaptive coarse-grained model for the aggregation of high-aspect-ratio carbon nanotubes. *J. Phys. Chem. C* **124**, 8359–8370 (2020).

[34] Reguero, V., Alemán, B., Mas, B. & Vilatela, J. J. Controlling carbon nanotube type in macroscopic fibers synthesized by the direct spinning process. *Chem. Mater.* **26**, 3550–3557 (2014).

[35] Motta, M. et al. The parameter space for the direct spinning of fibres and films of carbon nanotubes. *Physica E* **37**, 40–43 (2007).

[36] Upham, D. C. et al. Catalytic molten metals for the direct conversion of methane to hydrogen and separable carbon. *Science* **358**, 917–921 (2017). 29146810 10.1126/science.aao5023

[37] Temizel-Sekeryan, S., Wu, F. & Hicks, A. L. Global scale life cycle environmental impacts of single- and multi-walled carbon nanotube synthesis processes. *Int. J. Life Cycle Assess.* **26**, 656–672 (2021).

[38] Lee, D. et al. Ultrahigh strength, modulus, and conductivity of graphitic fibers by macromolecular coalescence. *Sci. Adv.* **8**, eabn0939 (2022). 35452295 10.1126/sciadv.abn0939 PMC9032978

[39] Huntsman Miralon product portfolio. *Hunstman* https://www.huntsman.com/products/detail/344/miralon (2024).

[40] Sherwin, E. D. et al. US oil and gas system emissions from nearly one million aerial site measurements. *Nature* **627**, 328–334 (2024). 38480966 10.1038/s41586-024-07117-5

[41] Jackson, R. B. et al. Increasing anthropogenic methane emissions arise equally from agricultural and fossil fuel sources. *Environ. Res. Lett.* **15**, 071002 (2020).

[42] Ocko, I. B. & Hamburg, S. P. Climate consequences of hydrogen emissions. *Atmos. Chem. Phys.* **22**, 9349–9368 (2022).

[43] Hanssen, S. V. et al. The climate change mitigation potential of bioenergy with carbon capture and storage. *Nat. Clim. Change* **10**, 1023–1029 (2020).

[44] Tong, D. et al. Committed emissions from existing energy infrastructure jeopardize 1.5 °C climate target. *Nature* **572**, 373–377 (2019). 31261374 10.1038/s41586-019-1364-3 PMC6697221

[45] Fulem, M. et al. Recommended vapor pressure and thermophysical data for ferrocene. *J. Chem. Thermodyn.* **57**, 530–540 (2013).

[46] Thiophene. *NIST* https://webbook.nist.gov/cgi/cbook.cgi?ID=C110021&Mask=4#Thermo-Phase (2023).

[47] Stallard, J. C. et al. The mechanical and electrical properties of direct-spun carbon nanotube mats. *Extreme Mech. Lett.* **21**, 65–75 (2018).

[48] Ashby, M. in *Materials Selection in Mechanical Design* 4th edn, 57–96 (Butterworth-Heinemann, 2011).

[49] Balasubramanian, M. *Composite Materials and Processing* (CRC Press, 2013).

