# Supplementary material for: Production of hydrogen and carbon nanotubes from methane using a multi-pass floating catalyst chemical vapour deposition reactor with process gas recycling
Source: Nat Energy. 2025 Dec 1;11(1):121–34. doi: 10.1038/s41560-025-01925-3 (PMC12851937; doi:10.1038/s41560-025-01925-3)
Supplement: Supplementary file 1 — Supplementary Tables 1 and 2, Supplementary Figs. 1–6, Supplementary Notes 1–7 and Supplementary References. [file 41560_2025_1925_MOESM1_ESM.pdf]

nature energy

Supplementary information https://doi.org/10.1038/s41560-025-01925-3

Production of hydrogen and carbon nanotubes from methane using a multi-pass floating catalyst chemical vapour deposition reactor with process gas recycling

In the format provided by the authors and unedited

Table of contents

2. Supplementary Table 1
2. Supplementary Table 2

3. Supplementary Figure 1
4. Supplementary Figure 2
5. Supplementary Figure 3
6. Supplementary Figure 4
7. Supplementary Figure 5
8. Supplementary Figure 6

9. Supplementary Note 1
10. Supplementary Note 2
12. Supplementary Note 3
14. Supplementary Note 4
15. Supplementary Note 5
17. Supplementary Note 6
18. Supplementary Note 7

19. Supplementary References

Supplementary Table 1

Supplementary Table 1 – Recipes used in this study: concentrated single-pass (SP), multi-pass (MP), and multi-pass with biogas (MPbio); dilute single-pass (SP2) and multi-pass (MP2). Recycled H_2 (yes/no) refers to whether all the H_2 carrier gas flows were recycled from the reactor exhaust (MP configuration), or whether exogenous hydrogen was supplied from a gas bottle (SP).

	H_2 (SCCM)						Ferrocene		Thiophene		
**Recipe**	**Bulk Carrier**	**Ferro-cene carrier**	**Thio-phene Carrier**	**Recy-cled H_2**	**CH_4 (SCCM)**	**CO_2 (SCCM)**	**Temp (°C)**	**Rate (mol h^{-1})**	**Temp (°C)**	**Rate (mol h^{-1})**	**Furnace (°C)**
**SP**	1400	180	60	No	160	0	105	0.0017	0.5	0.0046	1300
**MP**	1575	180	30	Yes	15	0	105	0.0017	0.5	0.0023	1300
**MPbio**	1575	180	30	Yes	15	7.5	105	0.0017	0.5	0.0023	1300
**SP2**	1566	103	31	No	100	0	90	0.00039	0.5	0.0024	1350
**MP2**	1672	103	15	Yes	10	0	90	0.00039	0.5	0.0012	1350

Supplementary Table 2 – Tortech Nanofibres Pilot Scale Recipes

Supplementary Table 2 – Recipes used by the Tortech Nanofibres pilot scale reactor. Tube diameter: 80 mm ID, 100 mm OD. Tube length: 1.8 m. Furnace set point: 1400 ºC.

$g\ h^{-1}$	Pilot SP 30 gph	Pilot MP 30 gph
**H_2**	150	0.0
**CH_4**	54	40
**Ferro**	9.3	9.3
**Thio**	6.0	3.0
**Total**	220	53

Supplementary Figure 1

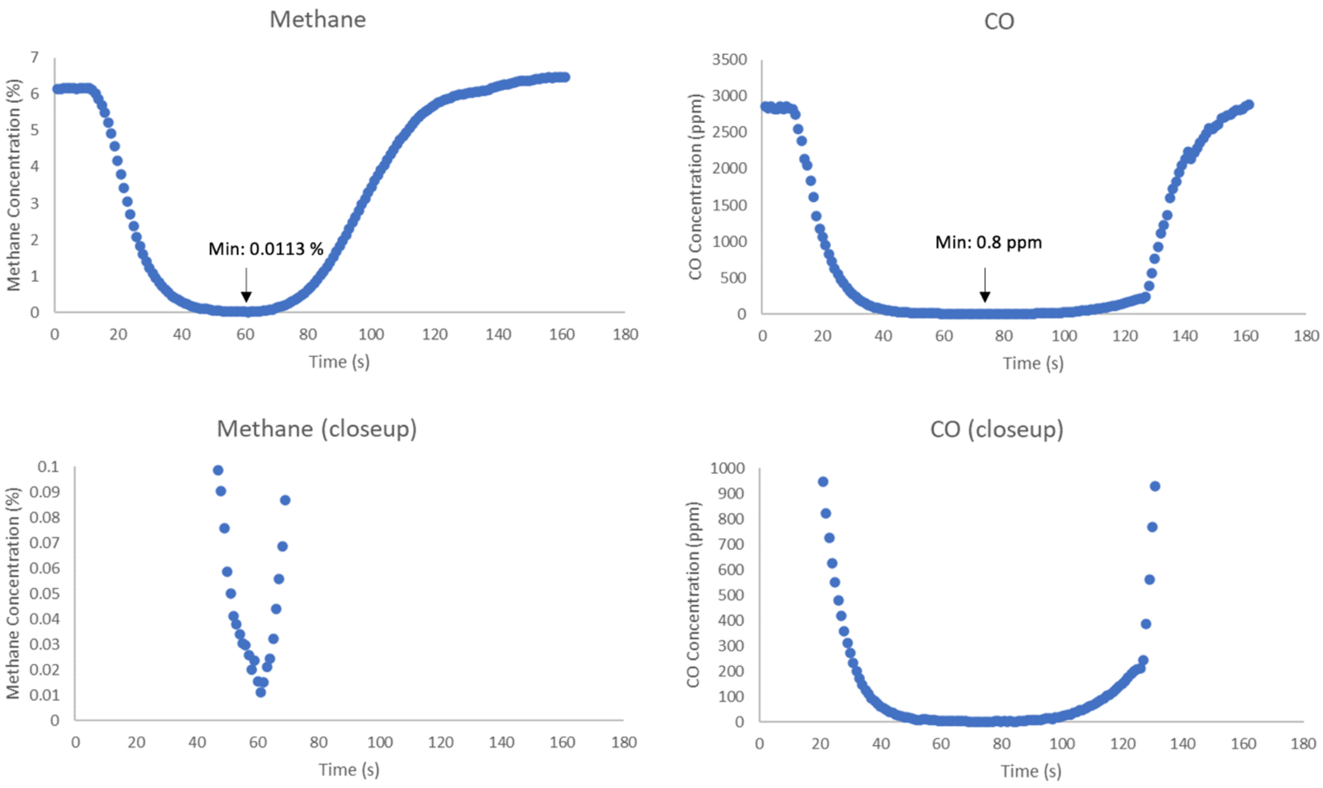

***Supplementary Figure 1** – Methane and carbon monoxide concentrations in reactor exhaust are reduced 2 – 3 orders of magnitude when fed through pressure swing absorption (PSA) columns containing powdered zeolite.* 1 SLPM of single-pass reactor exhaust was flowed through a column containing powdered zoelite, supplied by Amnis Pura. The exhaust initially contained 6 vol% methane and 3000 PPM CO. Within a minute of the exhaust flowing through the column, these concentrations reduced to 113 PPM and 0.8 PPM, respectively. The relatively large exhaust flow rate (~50x larger than MP exhaust) and small column used for this experiment meant the powder saturated quickly and impurity levels began to rise after 60 seconds (methane) and 90 seconds (CO). These initial experiments suggest that absorption columns could be used to reduce CH_4 and CO concentrations in the MP reactor exhaust down to ppm levels. Since these are the two primary impurities in the MP exhaust, this could enable hydrogen purification up to ~99% H_2.

Supplementary Figure 2

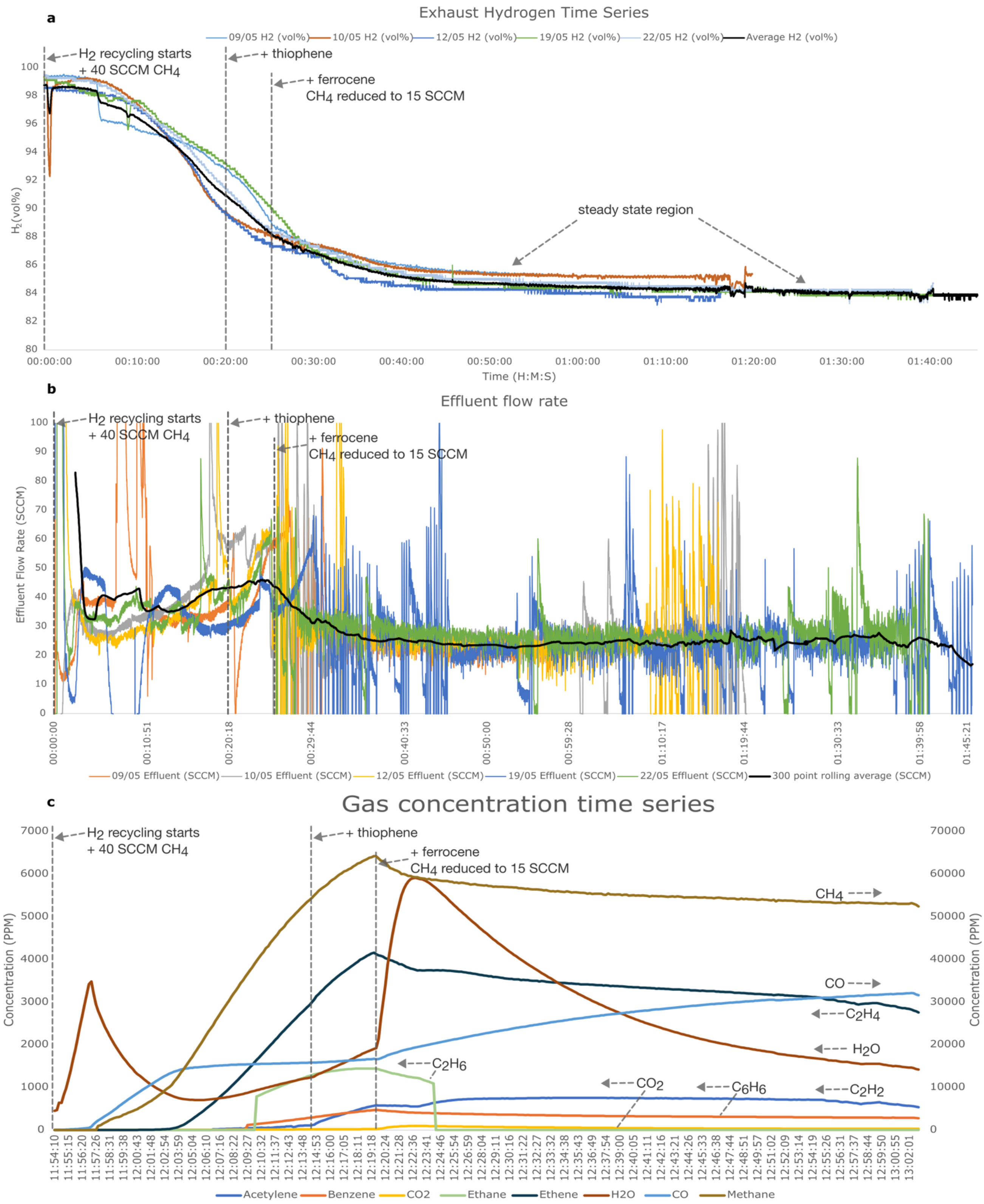

***Supplementary Figure 2** – Gas concentration time series showing start-up and evolution towards steady state. (a) Effluent hydrogen concentration, (b) effluent flow rate, and (c) other process gas species in the MP reactor. CH_4 and CO are plotted against the right axis in (c), other species are plotted against the left axis (arrows indicate the appropriate axis).*

Supplementary Figure 3

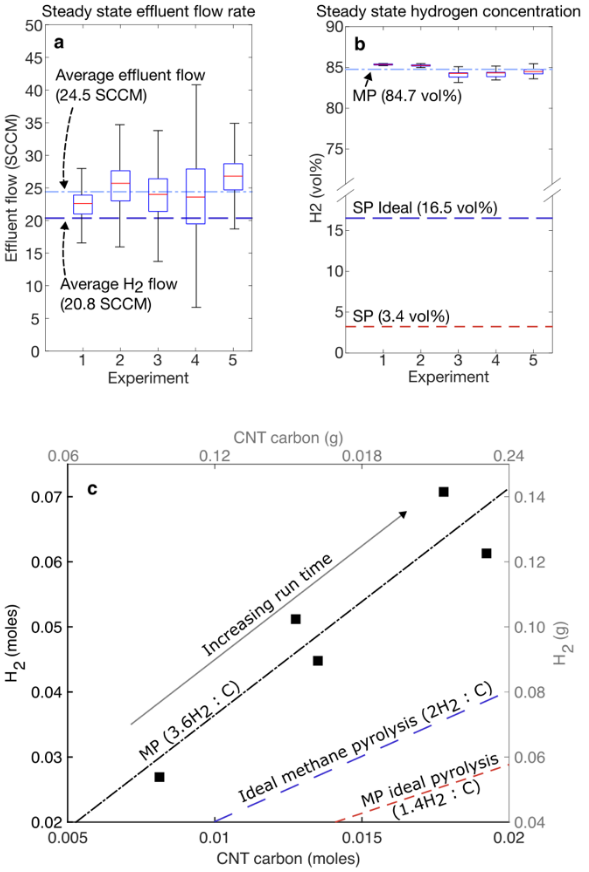

Supplementary Figure 3– Combined hydrogen and CNT production**.** (a) Steady-state flow rate of effluent gas leaving the MP reactor, and (b) concentration of hydrogen in the effluent gas, measured over 5 experiments. Box plot centerline displays the median value (50th percentile), the bounds of the box display the 25th (Q1) and 75th (Q3) percentile, and the whiskers extend to the most extreme values within 1.5 times the interquartile range above and below the box; values outside this range are considered outliers. The purple dot-dashed line in (a) shows the average effluent flow rate across experiments, the blue dashed line shows the average flow of H_2 within the effluent flow. The purple dot-dashed line in (b) shows the average hydrogen concentration across experiments. The red and blue dashed lines in (b) show the actual and ideal concentrations of H_2 produced inside the SP reactor, respectively. In (c), H_2 production is plotted against carbon production (in the form of CNTs). Experimental data from the MP reactor

Supplementary Figure 4

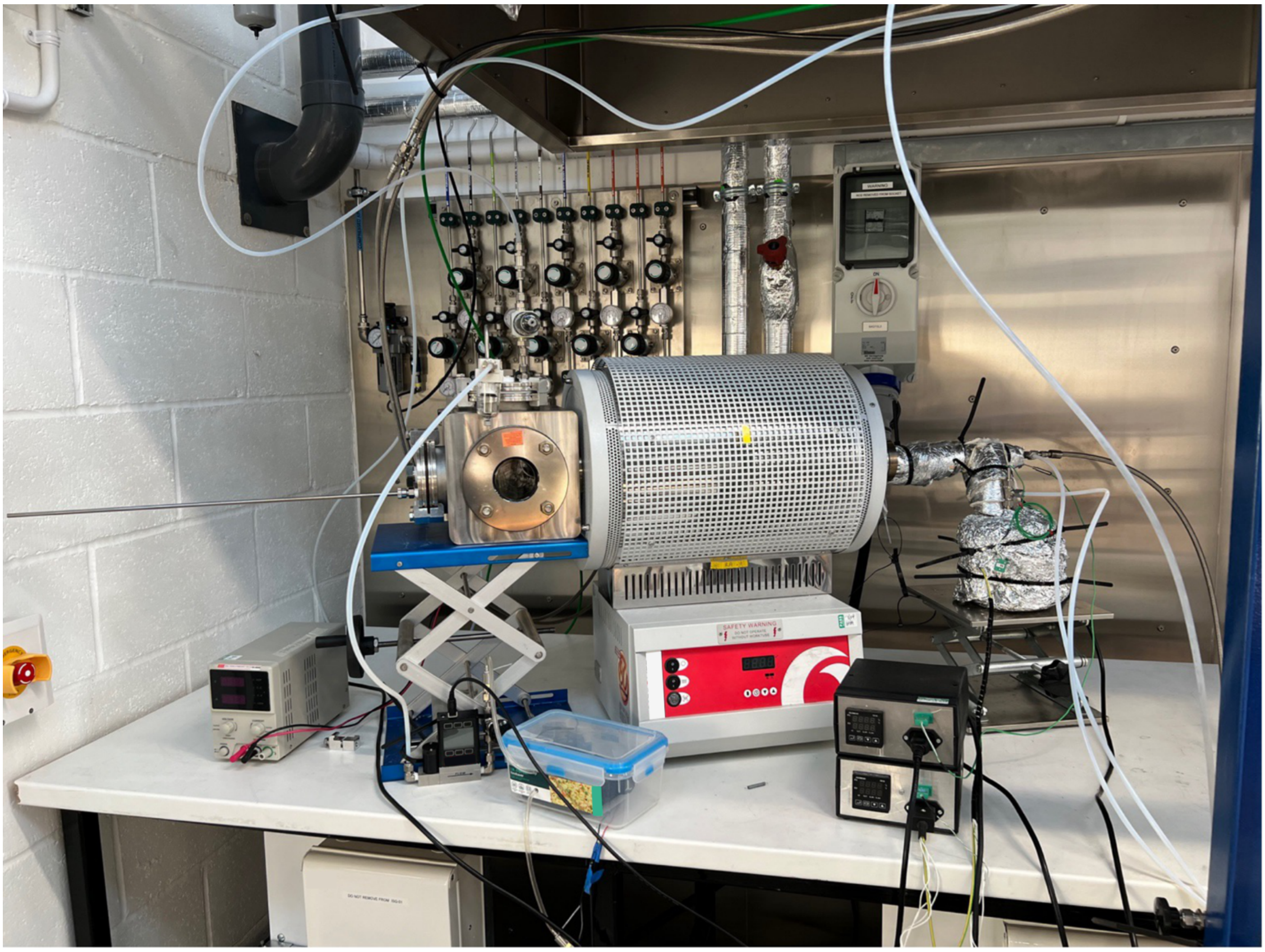

Supplementary Figure 4*– Reactor used in this study.* The catalyst dispensers are located on the right, with the tube furnace in the centre, and the collection chamber on the left. Gas dispensers on the wall supply a control tower (not shown) which controls flow rates and distributes gas to the catalyst dispensers and injector. Gas enters the tube furnace where the reaction takes place. CNTs and process gas leaves the tube furnace into the collection chamber, which contains an electric roller to collect carbon nanotubes. The “fishing rod” used to catch CNTs from the reaction tube and draw them onto the roller can be seen extending out from the left of the collection chamber. The recycle pump and MFC can be seen in the bottom of the image. These draw gas from the collection chamber and return it to the control tower, where the cycle starts again.

Supplementary Figure 5

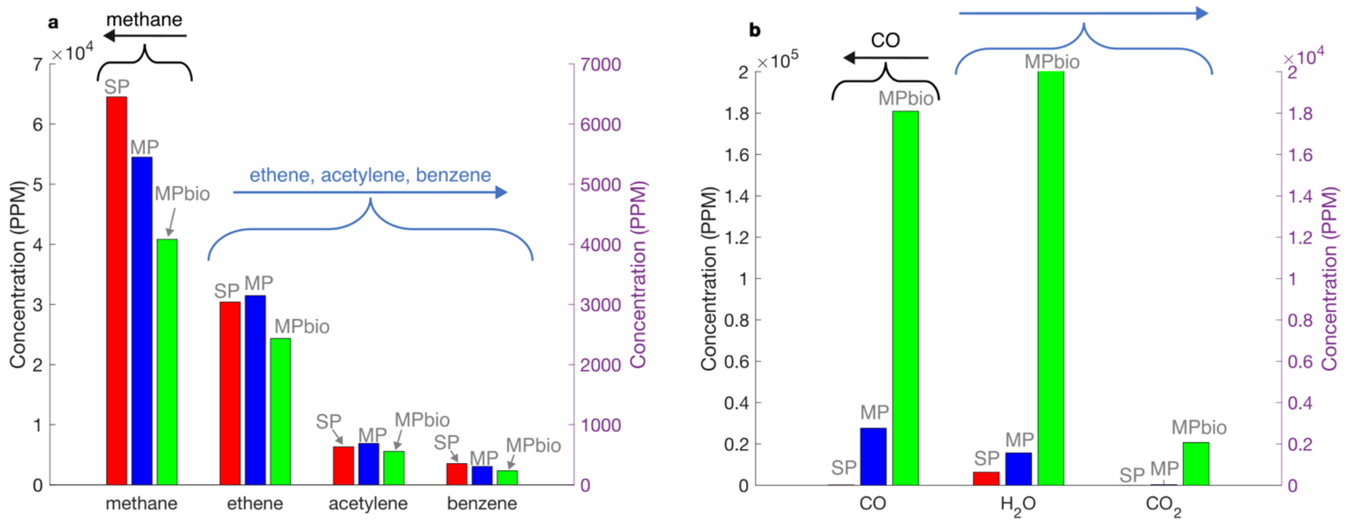

***Supplementary Figure 5** – Steady state concentrations of hydrocarbons and oxides in the concentrated single pass (SP), multi-pass (MP), and multi-pass with biogas (MPbio) process gas. (a) Hydrocarbon species and (b) oxides**,** measured using FTIR.*

Supplementary Figure 6

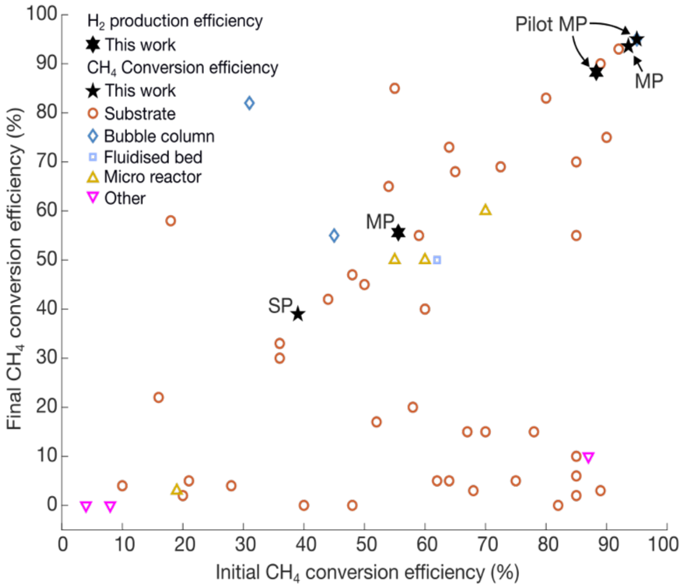

Supplementary Figure 6 – Methane conversion efficiency and hydrogen production efficiency (c) compared to other methane pyrolysis reactors using data presented by Fan et al. (2021) [1] and Qian et al. (2020) [2]. Many systems show a decline in CH_4 conversion as the reactor is fouled and the catalyst deactivated. In contrast, an FCCVD process that continuously removes carbon from the reactor as aerogel and replenishes the catalyst shows no decline in CH_4 conversion. The single-pass process, with CH_4 conversion of 27%, performs unfavourably even with this advantage. Additionally, the large consumption hydrogen carrier gas by the single pass process make hydrogen production impractical with this reactor. However, recycling the process gas in the multi-pass reactor increases CH_4 conversion to 92%: comparable to the best methane-pyrolysis reactors considered here. When considering the contribution of hydrogen from the other hydrocarbon precursors in the FCCVD process, along with losses, the overall hydrogen production efficiency of the multi-pass process is reduced to 54%; still comparable to many of the methane pyrolysis reactors reviewed here. The model of the pilot scale multi-pass process increases CH_4 conversion and overall hydrogen production efficiency to 88% and 95%, respectively.

Supplementary Note 1

Supplementary Figure 2a shows time series of the H_2 concentration measured in the effluent gas stream of the MP reactor. H_2 concentration begins near 100% because the reactor is purged with hydrogen before the experiment begins. The hydrogen concentration decreases for the first 20 – 30 minutes after ferrocene injection, before plateauing around 85 vol%. There is some variation between the time-evolution of the H_2 concentration in each experiment, but all experiments stabilise around 85 vol% H_2. The steady state region of an experiment is defined as the region where the hydrogen concentration is decreasing by less than 1% per hour.

Supplementary Figure 2b shows times series of the MP reactor effluent flow rate. The effluent flow rate varies somewhat up to the 25-minute mark, at which point ferrocene in injected to start the CNT synthesis process and within 10 minutes the effluent flow rate stabilises to around 25 SCCM, where it remains for the rest of each experiment. The peaks (both positive and negative) observed in the effluent flow rate are due to manually 'fishing' the aerogel out of the reactor when it breaks. The cause of such breaks is normally the aerogel mechanically adhering to the reaction tube and tearing. When this occurs, an Ultra-Torr fitting is loosened and a stainless-steel rod is inserted into the reaction tube to collect the aerogel and draw it out onto the roller, at which point normal collection resumes. This procedure results in an initial drop in effluent flow as the fitting is loosened, followed by a rebounding overshoot in flow rate once the fitting is tightened and sufficient pressure has been established inside the reactor to restart flow through the 1-way valve on the exhaust.

Supplementary Figure 2B shows the concentrations of other gas species in the MP process gas. Methane concentration rises to 60,000 PPM over the first 25 minutes of the experiment. It drops off after ferrocene is injected and then plateaus around 54,000 PPM. Concentration of other hydrocarbons – ethene, acetylene, and benzene – also increase at the start of an experiment and then plateau, though at an order of magnitude lower concentrations than methane. Ethane is measured early in the reaction, but its signal falls to zero shortly after ferrocene is injected.

H_2O concentration peaks when ferrocene is first injected into the reactor, suggesting the presence of water in the ferrocene pack, possible adsorbed onto the ferrocene. H_2O concentration then decreases asymptotically over the course of the experiment, plateauing around 1000 SCCM. CO concentration slowly rises over the course of the experiment. The authors believe that CO forms from the H_2O in the reactor and oxygen that enters the reactor via air leaking into the system around the low-pressure region up stream of the recycle pump, from impurities in the feed gasses, or from the etching the alumina (aluminium oxide) furnace tube. If a slow leak is present, oxygen will gradually be drawn into the reactor over the course of an experiment. It will react with the abundant carbon inside the reactor to form CO, which doesn't react (see S4 for discussion). If oxygen in the system is the result of an air leak, one would expect to find N_2 entering the system at four times the rate of O_2. This would result in approximately 5 vol% N_2 in the reaction mixture by the end of an experiment. However, the FTIR is unable to detect nitrogen, thus we can only infer its presence.

Supplementary Note 2

During steady-state operation, the MP reactor continuously converts CH_4 into H_2 gas and CNT aerogel. Steady state was achieved between 30 – 40 minutes after the start of an experiment, as shown in Supplementary Figure 3. Supplementary Figure 3a shows that the flow rate of effluent gas leaving the MP reactor was consistent across five experiments. The average effluent flow rate was 24.5 SCCM across the five experiments. This effluent flow was produced with an exogenous methane supply of 15 SCCM, meaning 9.5 SCCM of gas was produced inside the reactor. Applying the flow rate corrections described in the methods section yields and average flow rate of 22.5 SCCM. The range of effluent flow rates observed in each experiment is due to the need to manually "fish" the CNT sock from the furnace tube when it breaks, as described in Supplementary Note 2.

The effluent gas leaving the MP reactor contained mostly endogenous H_2 produced by the pyrolysis of methane. The box and whisker plots at the top of Supplementary Figure 3b show the median steady state H_2 concentration was consistent across all five experiments and averaged 84.7 vol%. The 24.5 SCCM effluent flow thus contains 20.8 SCCM of H_2, as indicated on figure Supplementary Figure 3a. The flow rate corrections for the effluent gas mixture, described in Supplementary Note 13, yield an effluent flow rate of 22.5 SCCM (19.1 SCCM H_2), which are presented in the main text.

Since there is no exogenous H_2 in the MP process, this result shows a net production of H_2 inside the reactor. A more detailed composition of the effluent stream, including the other species present, is given in Supplementary Figure 4. For comparison, the concentration of endogenous H_2 produced in the SP reactor is shown at the bottom of Supplementary Figure 3b. The SP reactor exhaust contains only 3.4 vol% endogenous H_2 because the large input of exogenous H_2 carrier gas heavily dilutes the pyrolysis reaction. Even in the ideal case, where 100% of the hydrocarbons in the SP process are pyrolyzed to produce H_2, this dilution would limit the endogenous H_2 concentration to 16.5 vol%, as shown in a blue dashed line in figure S3b. Removing the exogenous H_2 supply enables the MP process to produce a relatively concentrated stream (84.7 vol%) of endogenous H_2.

The pyrolysis of methane inside the MP reactor was very efficient; however, losses inside the reactor resulted in less carbon being collected in the form of CNT mat that predicted by the ideal pyrolysis chemistry. The experimental data in Supplementary Figure 3c, plotted in black squares, shows an increase in production of both hydrogen and CNT carbon as run time increases, following a linear trend as one would expect from the ideal pyrolysis reaction. The ideal chemistry of methane pyrolysis in equation (1) of the main text yields a molar ratio of 2:1 (H_2:C), plotted as a blue dashed line. The ideal pyrolysis reaction inside the MP process, accounting for the ferrocene and thiophene precursors, is given by

$$\begin{aligned} \mathrm{CH_4}(g) + 0.04\ \mathrm{C_{10}H_{10}Fe}(g) + 0.06\ \mathrm{C_4H_4S}(g) \\ \rightarrow 2.33\ \mathrm{H_2}(g) + 1.65\ \mathrm{C}(s) + 0.04\ \mathrm{Fe}(s) + 0.06\ \mathrm{S}(s), \end{aligned}$$

where the molar rates of reactants come from the recipe given in Supplementary Table 1. The contributions of ferrocene and thiophene to the pyrolysis reaction yields a H_2:C ratio of 1.4, the plotted in red dashes at the bottom right of Supplementary Figure 3c. The slope of H_2:CNT production measured experimentally in the MP reactor is 3.6:1, plotted in a black dot-dashed line. Compared to ideal MP pyrolysis, the experimental MP process produces a higher ratio of H_2 to carbon. This is due to carbon losses – carbon that has undergone pyrolysis but is lost inside the reactor such that it contributes to hydrogen production but not CNT production. Clearly, the high level of loss in the MP reactor causes it to operate far from its ideal chemistry. This impact of solid loss on the process, CNT product and its implications for scaleup as discussed in the main text.

Supplementary Note 3

Supplementary Figure 5a compares the steady state concentrations of hydrocarbons in the exhaust (SP) and recycled (MP, MPbio) gas streams of each process, measured using FTIR. Ethene, acetylene, and benzene, formed via methane pyrolysis, are observed in similar concentrations in all processes (3000, 600, 300 PPM, respectively), while no ethane was detected during steady state operation in any configuration. The concentration of unreacted methane in the exhaust of the SP process is 65,000 PPM dropping to 54,000 PPM in the MP process and 41,000 PPM in the MPBio process.

Supplementary Figure 5b shows the steady state concentration of oxide impurities, CO, H_2O, and CO_2 in each process. Oxide concentrations are very low in the SP process. The MP process shows higher levels of oxides: 28,000 PPM CO and 1600 PPM H_2O. The presence of these oxygen-containing species suggests the ingress of air; a likely leak path is through the plumbing exposed to low-pressure upstream of the recycle pump. Other explanations are oxide contaminants in the feed gasses, or etching of oxygen from the alumina (aluminium oxide) furnace tube: oxides from these sources would be vented from the reactor after each pass in SP configuration, but in MP configuration they would accumulate inside the reactor resulting in the higher concentrations shown in Supplementary Figure 5b.

The MPBio process shows very high concentration of oxygen-containing species in Supplementary Figure 5b, particularly CO (180,000 PPM) and H_2O (11,000 PPM). The oxygen is provided by the CO_2 in the biomethane precursor, which acts as a mild oxidising agent inside the reactor. A water-gas-shift reaction could convert

$$CO_2 + H_2 \rightarrow CO + H_2O.$$

Such a reaction would result in 1:1 ratios of H_2O and CO, yet Supplementary Figure 5b shows 16x more CO than H_2O in the MPbio process gas. This suggests the CO_2 is oxidisng other carbon species to create additional CO, hence CO_2 is believed to act as a carbon sink in the process that interferes with the synthesis of CNTs.

It should be noted that, in the case of the MP and MPbio processes, the gas mixtures measured here are recycled and re-injected into the reactor. As a result, 10^3 PPM levels of methane pyrolysis products and H_2O are injected into the MP process along with 10^4 PPM levels of CO. In the MPbio process, concentrations of H_2O and CO at the injector will be approximately 10^4 PPM and 10^5 PPM, respectively. The MPbio process also sees 10^3 PPM levels of CO_2, due to the impurities in the carbon source and the residual CO_2 recycled in this process. The addition of these species is likely to cause the differences in process performance and material quality highlighted in Fig. 5 in the main text.

These differences between material produced in single-pass and multi-pass reactors may result from the injection of hydrocarbon pyrolysis products (including acetylene) and H_2S into the reactor along with methane, thiophene, and ferrocene. Several studies suggest that acetylene is the direct precursor to CNT growth: methane first decomposes into acetylene before catalytically growing CNTs [7-9]. According to the HACA mechanism, acetylene is also the precursor to soot formation [10,11]. Injection of acetylene directly into the reactor is thus likely to influence the growth of CNTs and

promote the formation of amorphous carbon. Furthermore, methane thermally decomposes above 1000 ºC while ferrocene decomposes around 500 ºC [12] so iron particle formation can occur before methane has cracked to provide acetylene for CNT growth. This provides time for iron clusters to coalesce into the large particles observed in the SP material. Injecting acetylene directly into the MP reactor means CNT growth can occur as soon as catalyst particles nucleate, potentially explaining the higher growth rate, lower iron content and smaller iron particles observed in the MP material [5]. It is unclear what role H_2S plays in the FCCVD process, but its injection into the MP process may also contribute to differences in CNT growth. For example, thiophene decomposes at higher temperature (~1000 ºC) than ferrocene [12], but there is evidence that having sulfur available early in the reactor can reduce iron particle coarsening [13].

Supplementary Note 4

The pilot-scale MP reactor considered here produces CNTs and hydrogen at a rate of 30 g h^{-1} and 9.4 g h^{-1} respectively, and consumes methane at a rate of 40 g h^{-1}. If such reactors were to supply 100 Mt of hydrogen (today's global hydrogen production and 1/4 of predicted demand in 2050 [14]) over 1 billion such reactors would be needed. Clearly, this is not a feasible number and the productivity of FCCVD reactors must be increased if they are to play a significant role in future hydrogen production. Fortunately, Huntsman Corporation have developed an FCCVD plant capable of producing 30 t(CNT) y^{-1} and plan to have a 4 kt(CNT) y^{-1} plant online by 2026, with longer-term goals to develop 1 Mt(CNT) y^{-1} plant[15-17]. Assuming these plants produce CNTs and hydrogen in the same proportion as the pilot plant studied here, the 1 Mt(CNT) plant would produce 330 kt(H_2) annually, requiring 300 plants to supply 100 Mt(H_2). The process would consume 400 Mt(CH_4) y^{-1}, equal to 16% of the world's natural gas production [18]. Diverting this natural gas away from combustion applications would mitigate 1.1 Gt(CO_2) emissions. The process would also produce 300 Mt(CNT) y^{-1}, equivalent to 18% of the world's steel production [18]. If these CNTs were used to replace CO_2 intensive materials like steel, a further cascade of emissions reductions could be achieved. Even if the CNT product was not considered and turquoise hydrogen used in place of today's hydrogen, produced primarily by steam methane reforming and contributing 830 Mt annual CO_2 emissions, the climate impact would be significant [1,19].

Supplementary Note 5

The recipes used in this study are presented in Supplementary Table 1. Bulk carrier gas refers to carrier gas mixed with the reactants in the injector and used to dilute the reaction mixture to control side reactions like soot formation. Ferrocene carrier gas was flowed through the ferrocene sublimation pack to collect ferrocene vapor and transport it into the reactor. Similarly, thiophene carrier gas was routed through the thiophene pack to collect thiophene vapor and transport it into the furnace. In the single-pass (SP) configuration, carrier gasses were supplied by a hydrogen gas bottle and were vented to exhaust after a single pass of the furnace. In the multi-pass (MP) configuration, during steady state operation, the carrier gasses were supplied from the reactor exhaust via a pump and no exogenous hydrogen was supplied from the gas bottle. A detailed schematic and description of the reactor is given in the Methods section.

Recipes were changed as little as possible between reactor configurations to provide the best comparison of the processes. The ferrocene pack, thiophene pack, and furnace set point were maintained at the same temperature between SP, MP and MPbio experiments, and SP2 and MP2 experiments, as was the ferrocene flow rate. Ferrocene undergoes complete thermal decomposition inside a ~1300 °C reactor and its contributions to the reaction – iron catalyst particles – are not recycled in the MP process. Instead, iron particles are collected with other solid products or filtered from the exhaust gasses with particle filters. Iron is therefore not recycled along with the process gasses, so the same ferrocene input is needed in both SP and MP operation. Conversely, thiophene contributes sulfur to the reactor, which is either bound into iron catalyst particles or forms H_2S gas. H_2S is recycled in the MP process and can thus contribute sulfur to the reaction on multiple passes. The thiophene input is halved in the multi-pass processes compared to the SP process because this was found to produce similar CNT spinning conditions. The authors acknowledge that further optimisation of the thiophene input could yield better results in a MP process. Like thiophene, the methane input in multi-pass processes was reduced compared to the SP process, otherwise excessive methane accumulated inside the reactor causing sooting and preventing continuous aerogel collection. This is because unreacted hydrocarbons are recycled with the process gas and can thus accumulate and react on multiple passes of the reactor. The total flow rate was kept at 1800 SCCM in all configurations except MPbio, where the total flow rate was increased to 1807.5 SCCM due to the addition of the CO_2 impurity. The reasons for adding the CO_2 in addition to other the other precursors is due to its oxidising and carbon-leaching behaviour discussed in section S4.

The SP2 and MP2 recipes were modified to produce CNTs of higher quality, particularly in terms of Raman G/D ratio and electrical conductivity. This was achieved primarily by reducing precursor concentrations [3,4] and increasing the furnace set point from 1300 ºC to 1350 ºC. Reducing the amount of carbon in the reactor (primarily by reducing methane input) helps to suppress processes like soot formation and amorphous/ graphitic carbon deposition on the surface of nanotubes [3]. These impurities interfere with the bundle and aerogel structure of the CNTs, generally resulting in worse properties. They also tend to increase the number of defect sites, resulting in a stronger

Raman D peak and worse CNT properties. Reducing the amount of iron by reducing ferrocene concentration helps to reduce the agglomeration of iron nanoparticles into large, inactive particles. Such large particles add iron impurity to the aerogel without contributing to CNT growth and tend to become encapsulated in graphitic carbon shells which increases the amount of non-CNT carbon impurity in the aerogel. Reducing sulfur in the reactor by diluting thiophene improves the crystallinity (G/D ratio) of carbon nanotubes and reduces the number of impurities in the aerogel [6]. The result of these changes was to increase electrical conductivity 10-fold and Raman G/D ratio 4-fold as shown in Fig. 4 of the main text.

However, reducing precursor concentrations also reduces the productivity and conversion efficiency of the reactor. Attempts to improve CNT properties in the literature are often accompanied by a reduction in reactor productivity [3]. The same effects were observed here, with ~50% reduction in CNT mass production and a similar reduction in yield and efficiency observed between the original and modified recipes.

Supplementary Note 6

Once clean, the reactor was sealed and heated up under a flow of air. Once hot, the reactor was purged with argon to displace the air, and then purged with hydrogen until it contained >97% H_2 atmosphere. During the H_2 purge, the ferrocene delivery system, thiophene delivery system, CH_4 lines and recycle lines were flushed with hydrogen for approximately 10 minutes to remove air, moisture, and other contaminants from the lines.

In multi-pass configuration, once a >97% H_2 atmosphere was achieved inside the reactor, the recycle MFC was set to meter 1760 SCCM H_2 and the recycle pump was turned on. The exogenous H_2 supply was closed with a tap and from this point onward the reactor received no exogenous hydrogen for the course of an experiment. An additional flow of 40 SCCM methane was injected into the reactor, giving a total flow rate of 1800 SCCM. This condition was maintained for 20 minutes to allow CH_4 to build up to a sufficient concentration. Supplementary Figure2 shows that the hydrogen concentration dropped to about 90% in this time, while methane concentration rose to about 60,000 PPM. At the 20 minute mark, 30 SCCM of H_2 was flowed through the thiophene pack to introduce thiophene into the reactor, the total flow into the reactor was maintained at 1800 SCCM. This condition was maintained for 5 minutes so thiophene/ sulfur compounds could accumulate inside the reactor. 25 minutes after the recycle loop was started, methane was reduced to 15 SCCM and H_2 was flowed through the ferrocene pack at 180 SCCM while the total flow into the reactor was maintained at 1800 SCCM. The addition of ferrocene initiated the CNT growth process and within a few minutes aerogel could be extracted from the end of the furnace tube and wound onto the roller. 20 - 30 minutes after ferrocene injection the process gas concentrations stabilised at their steady state values (see Supplementary Figure 2). Steady state was defined as the portion of an experiment where the effluent H2 concentration was decreasing by less than 1% per hour. The experiment would continue until the aerogel could no longer be collected. At this point, reactants would be shut down and the reactor and recycle line would be flushed with exogenous H_2, before being purged with argon and then air, as the reactor was cooled down.

The startup procedure in single-pass configuration was simpler. Once a >97% H_2 atmosphere was created inside the reactor, 160 SCCM methane would be flowed for 5 minutes, 60 SCCM H_2 would be flowed via the thiophene pack for 2 minutes, and then 180 SCCM H_2 would be flowed via the ferrocene pack to start the reaction. Like the multi-pass process, total flow was kept at 1800 SCCM throughout the experiment, and the reactor was run until aerogel could no longer be collected, at which point the same shut down and purge procedures were followed.

Supplementary Note 7

Alicat MFCs and MFMs measure the gas temperature and the pressure drop across a constriction. The volumetric flow rate, V, is calculated using the expression:

$$V = \frac{K\Delta P}{\mu},$$

where ΔP is the measured pressure drop and μ is the gas viscosity, and K is defined as

$$\frac{4\pi r}{8L},$$

where r and L relate to the geometry of the constriction in the mass flow meter, thus K remains constant in this analysis [20]. The volumetric flow rate is converted into a standard volumetric flow rate (the equivalent volumetric flow rate at STP conditions) using the relation between the gas viscosity μ and temperature (T). Standard volumetric flow rate is related to the mass flow rate (m) of the fluid by the density at STP according to the following equation:

$$m = \rho_{STP} V_{STP}$$

where subscript STP denotes STP conditions. This method of mass flow measurement results in an error if the gas viscosity differs from the one the MFC is programmed to use. For example, if a mixture is present rather than a pure gas, as is the case for the hydrogen stream leaving the multi-pass reactor (84.7 vol% H_2), the mixture viscosity will be different to that of pure H_2 causing an error in the calculated mass flow rate. The multi-pass effluent mass flow rates presented here were corrected using the composition of the effluent gas, measure by FTIR and MS, to calculate the viscosity of the mixture according to Graham's model for partial viscosity sums and individual gas viscosity values from NIST [21].

Supplementary References

1. Fan, Z., Weng, W., Zhou, J., Gu, D. & Xiao, W. Catalytic decomposition of methane to produce hydrogen: A review. *Journal of Energy Chemistry* **58**, 415–430 (2021).
2. Qian, J. X. *et al.* Methane decomposition to pure hydrogen and carbon nano materials: State-of-the-art and future perspectives. *International Journal of Hydrogen Energy* **45**, 15721–15743 (2020).
3. Reguero, V., Alemán, B., Mas, B. & Vilatela, J. J. Controlling Carbon Nanotube Type in Macroscopic Fibers Synthesized by the Direct Spinning Process. *Chem. Mater.* **26**, 3550–3557 (2014).
4. Motta, M. *et al.* The parameter space for the direct spinning of fibres and films of carbon nanotubes. *Physica E: Low-dimensional Systems and Nanostructures* **37**, 40–43 (2007).
5. Lee, S.-H. *et al.* Synthesis of carbon nanotube fibers from carbon precursors with low decomposition temperatures using a direct spinning process. *Carbon* **124**, 219–227 (2017).
6. Paukner, C. & Koziol, K. K. K. Ultra-pure single wall carbon nanotube fibres continuously spun without promoter. Sci Rep 4, 3903 (2014).
7. Eres, G. *et al.* Model for Self-Assembly of Carbon Nanotubes from Acetylene Based on Real-Time Studies of Vertically Aligned Growth Kinetics. *J. Phys. Chem. C* **113**, 15484–15491 (2009).
8. Eres, G. *et al.* Molecular Beam-Controlled Nucleation and Growth of Vertically Aligned Single-Wall Carbon Nanotube Arrays. *J. Phys. Chem. B* **109**, 16684–16694 (2005).
9. Zhong, G. *et al.* Acetylene: A Key Growth Precursor for Single-Walled Carbon Nanotube Forests. *J. Phys. Chem. C* **113**, 17321–17325 (2009).
10. Parker, D. S. N., Kaiser, R. I., Troy, T. P. & Ahmed, M. Hydrogen Abstraction/Acetylene Addition Revealed. *Angewandte Chemie International Edition* **53**, 7740–7744 (2014).
11. Johansson, K. O., Head-Gordon, M. P., Schrader, P. E., Wilson, K. R. & Michelsen, H. A. Resonance-stabilized hydrocarbon-radical chain reactions may explain soot inception and growth. *Science* **361**, 997–1000 (2018).
12. Hoecker, C. *et al.* Catalyst nanoparticle growth dynamics and their influence on product morphology in a CVD process for continuous carbon nanotube synthesis. *Carbon* **96**,116–124 (2017)
13. Lee, S.-H., Park, J., Kim, H.-R., Lee, J. & Lee, K.-H. Synthesis of high-quality carbon nanotube fibers by controlling the effects of sulfur on the catalyst agglomeration during the direct spinning process. *RSC Adv.* **5**, 41894–41900 (2015).
14. IEA. Global hydrogen demand in the Net Zero Scenario, 2022-2050 – Charts – Data & Statistics. *IEA* https://www.iea.org/data-and-statistics/charts/global-hydrogen-demand-in-the-net-zero-scenario-2022-2050 (2023).
15. Huntsman. Huntsman Miralon Product Portfolio. *Huntsman Corporation* https://www.huntsman.com/products/detail/344/miralon (2024).
16. Dave Gailus. Structural, High Value Carbon and Hydrogen from Natural Gas (Huntsman). (2021).

17. Gailus, D. Structural, High Value Carbon and Hydrogen from Natural Gas (Huntsman). (2022).
18. Pasquali, M. & Mesters, C. We can use carbon to decarbonize—and get hydrogen for free. *Proceedings of the National Academy of Sciences* **118**, e2112089118 (2021).
19. Diab, J., Fulcheri, L., Hessel, V., Rohani, V. & Frenklach, M. Why turquoise hydrogen will Be a game changer for the energy transition. *International Journal of Hydrogen Energy* **47**, 25831–25848 (2022).
20. Alicat Scientific. Laminar Differential Pressure Flow Meter - Operating Principle. *Alicat Scientific* https://www.alicat.com/choosing-an-instrument/theory-of-operation-laminar-differential-pressure-flow-measurement/ (2021).
21. NIST. Thermophysical Properties of Fluid Systems. *NIST Chemistry WebBook* https://webbook.nist.gov/chemistry/fluid/ (2023).
