# Supplementary figures and images for: Production of hydrogen and carbon nanotubes from methane using a multi-pass floating catalyst chemical vapour deposition reactor with process gas recycling
Source: Nat Energy. 2025 Dec 1;11(1):121–34. doi: 10.1038/s41560-025-01925-3 (PMC12851937; doi:10.1038/s41560-025-01925-3)

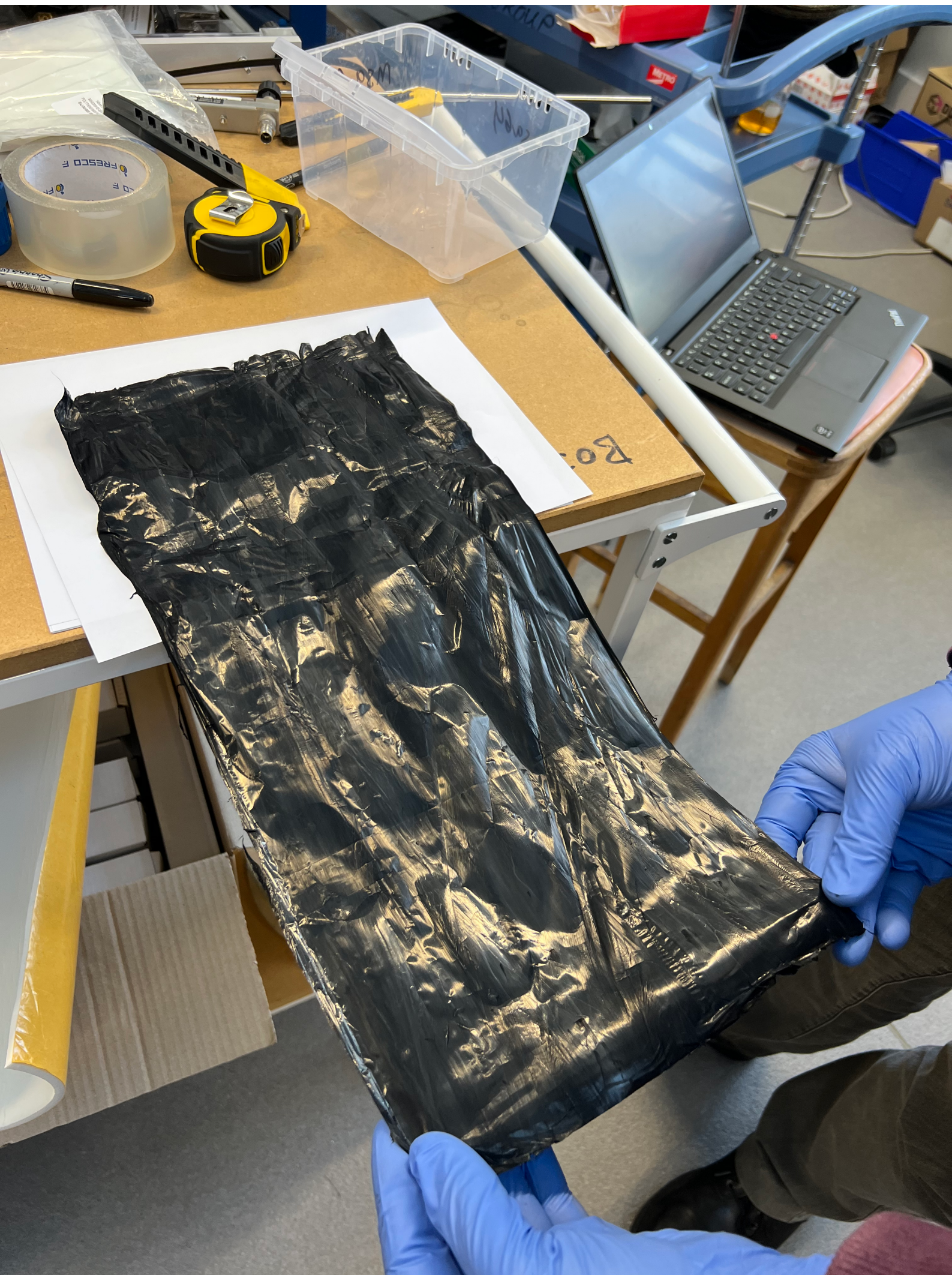

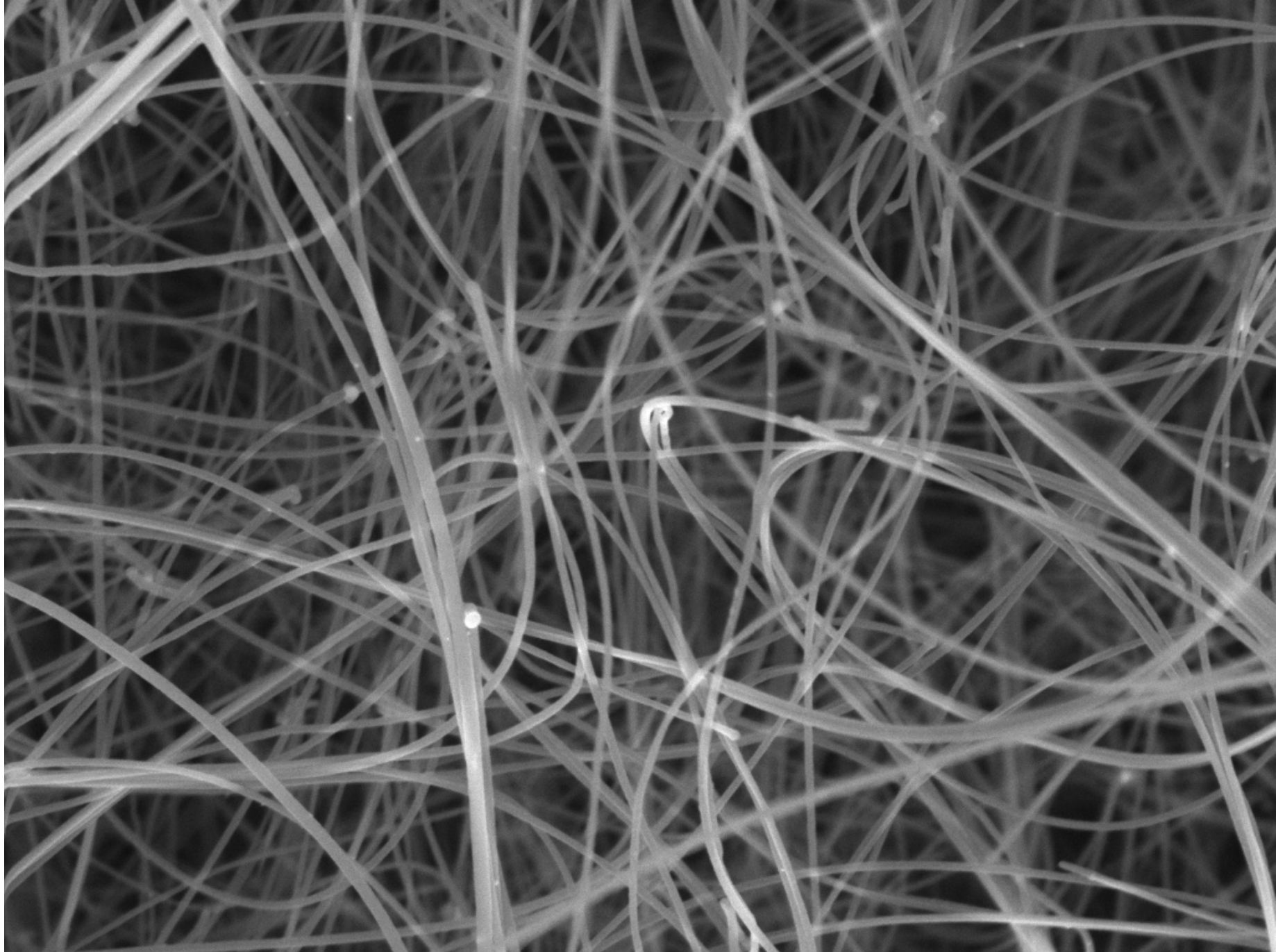

Stage Tilt: 0.0°

WD: 5.25 mm

SEM MAG: 55.4 kx

View field: 5.00  $\mu\text{m}$

Det: In-Beam SE

SEM HV: 5.0 kV

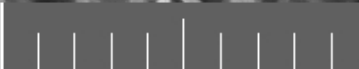

1  $\mu\text{m}$

MIRA3 TESCAN

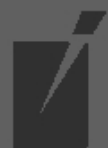

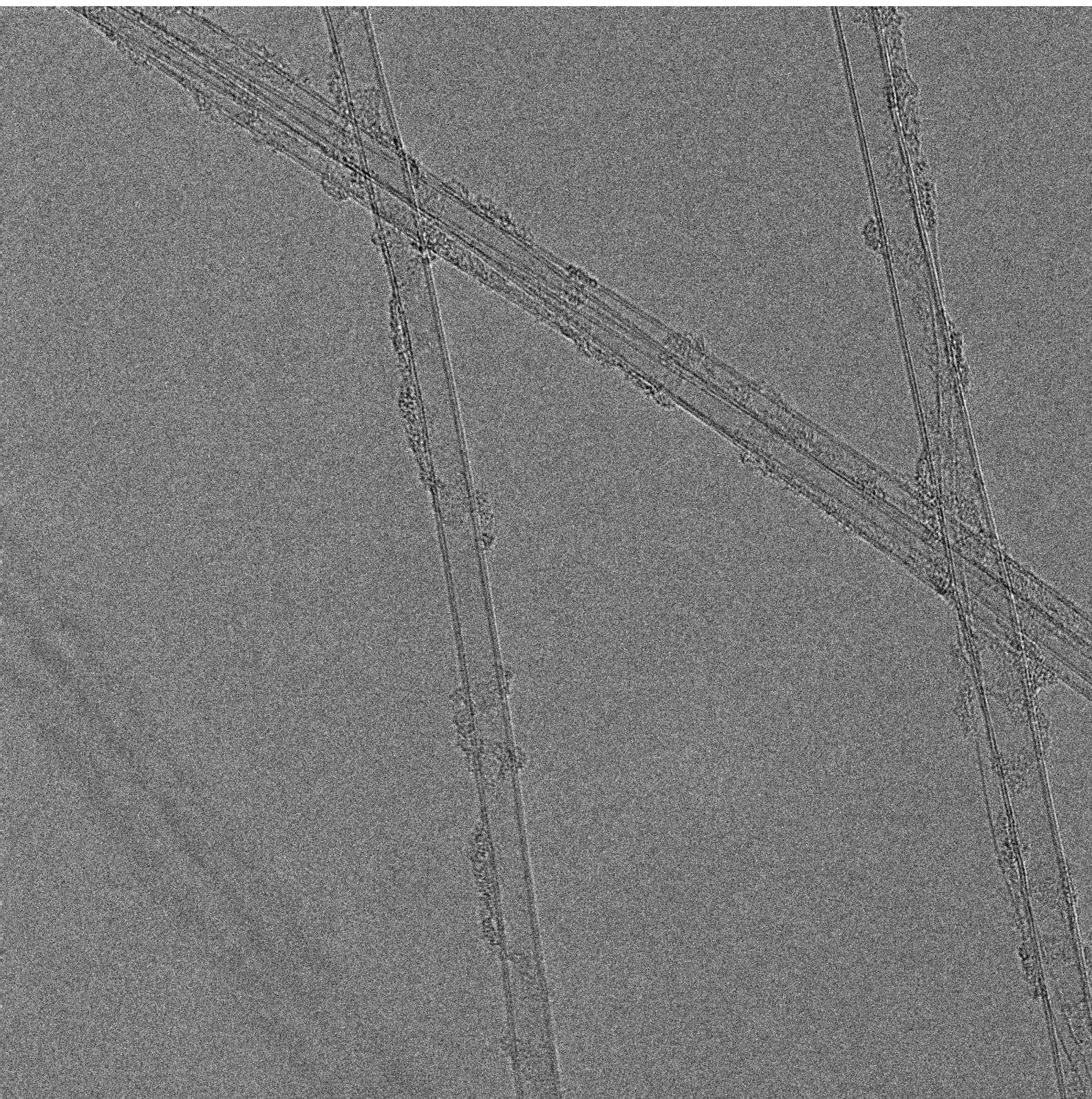

— 20 nm

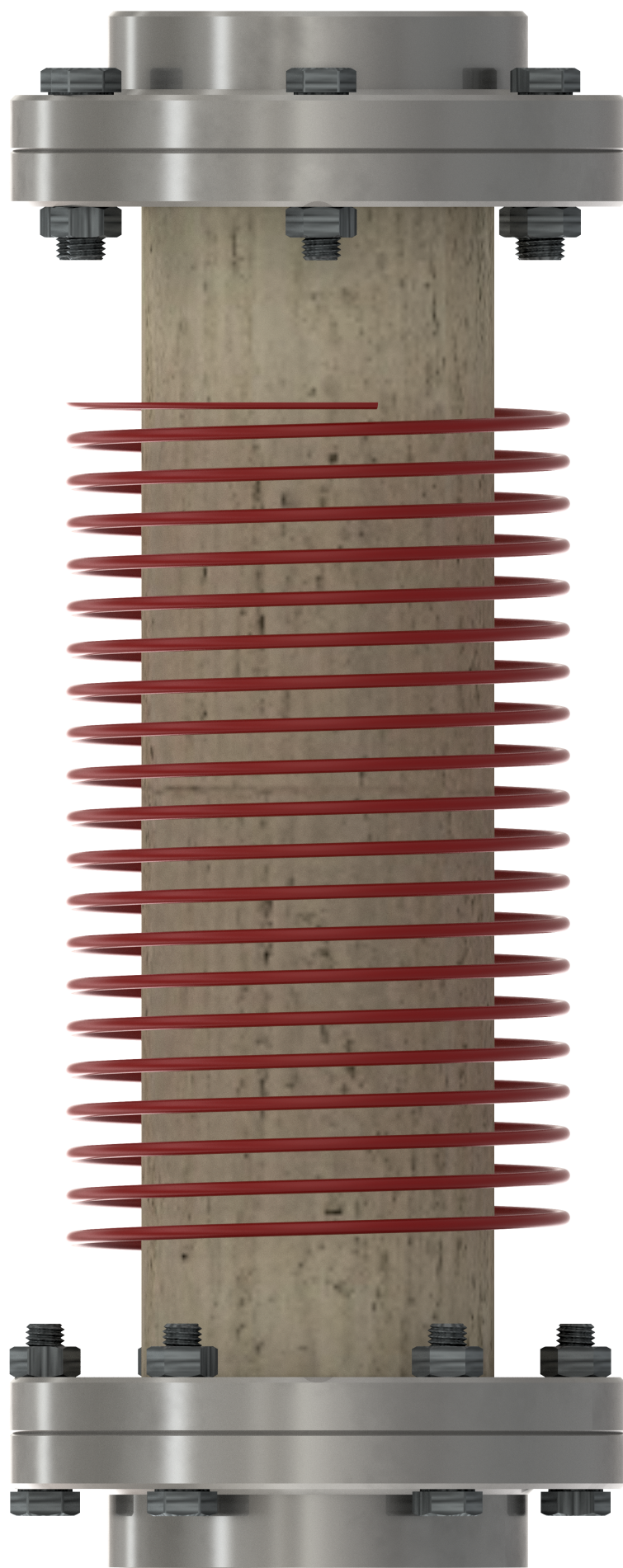

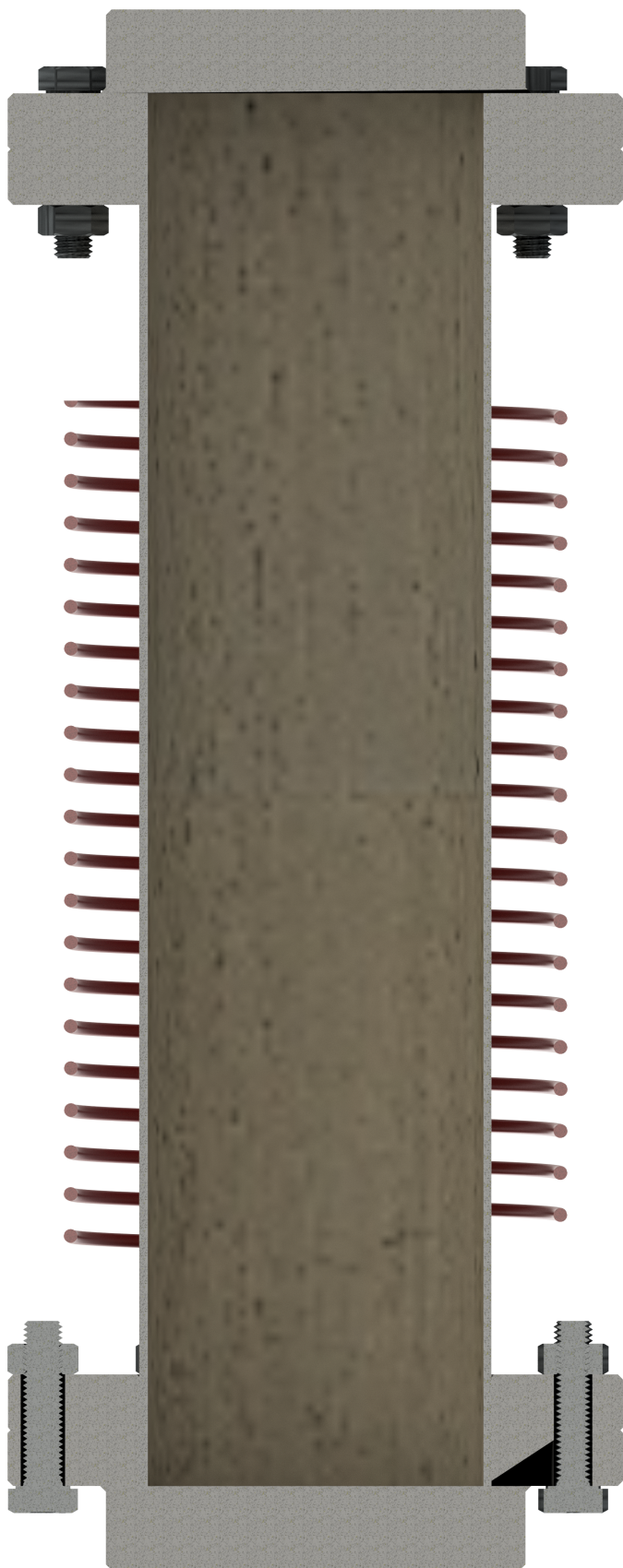

Supplement: Supplementary file 4 — Unprocessed images used in the ‘metre’, ‘micrometre’, ‘nanometre’ and renders of the reactor used in Fig. 1a. [file 41560_2025_1925_MOESM4_ESM.pdf]

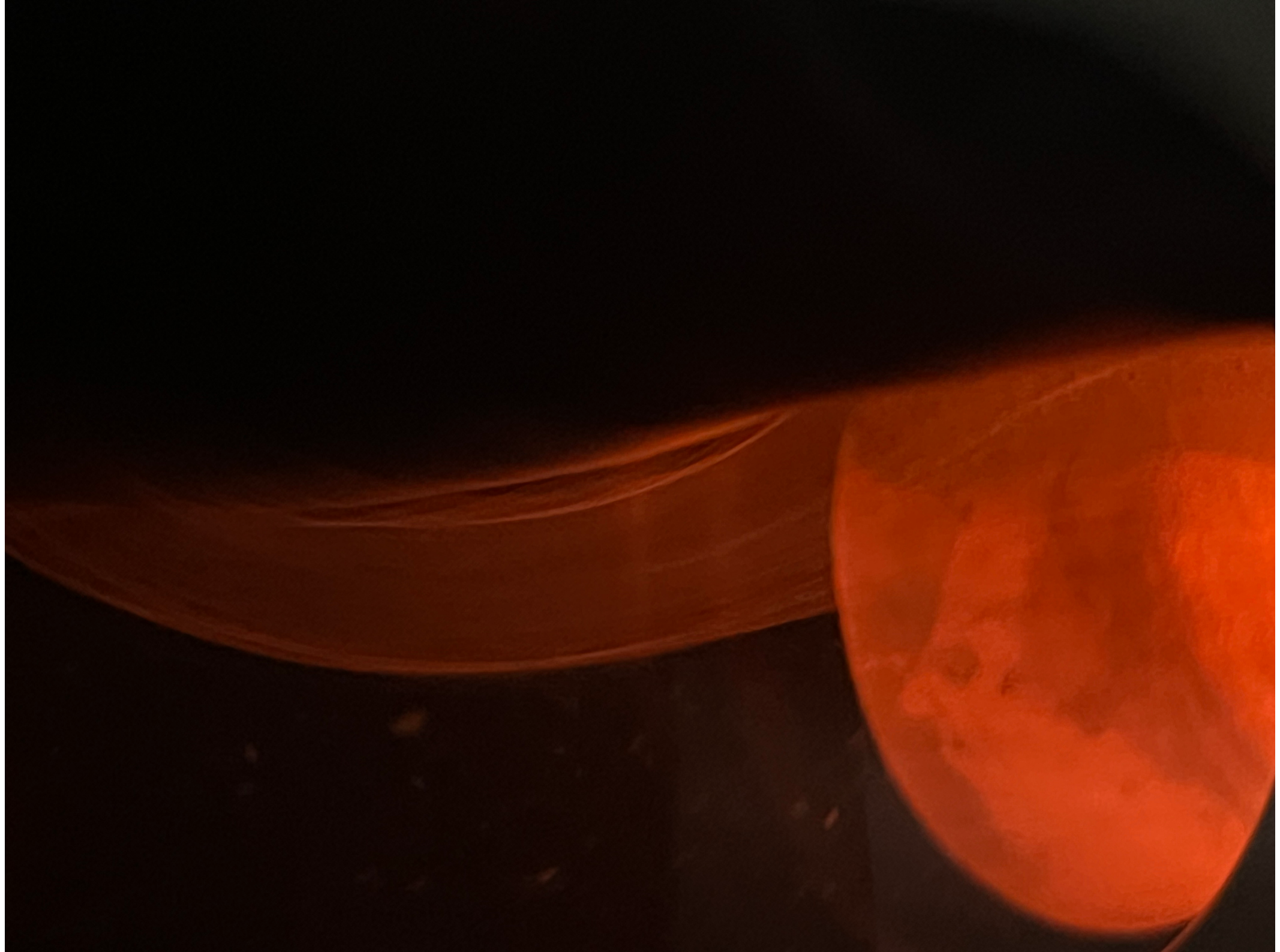

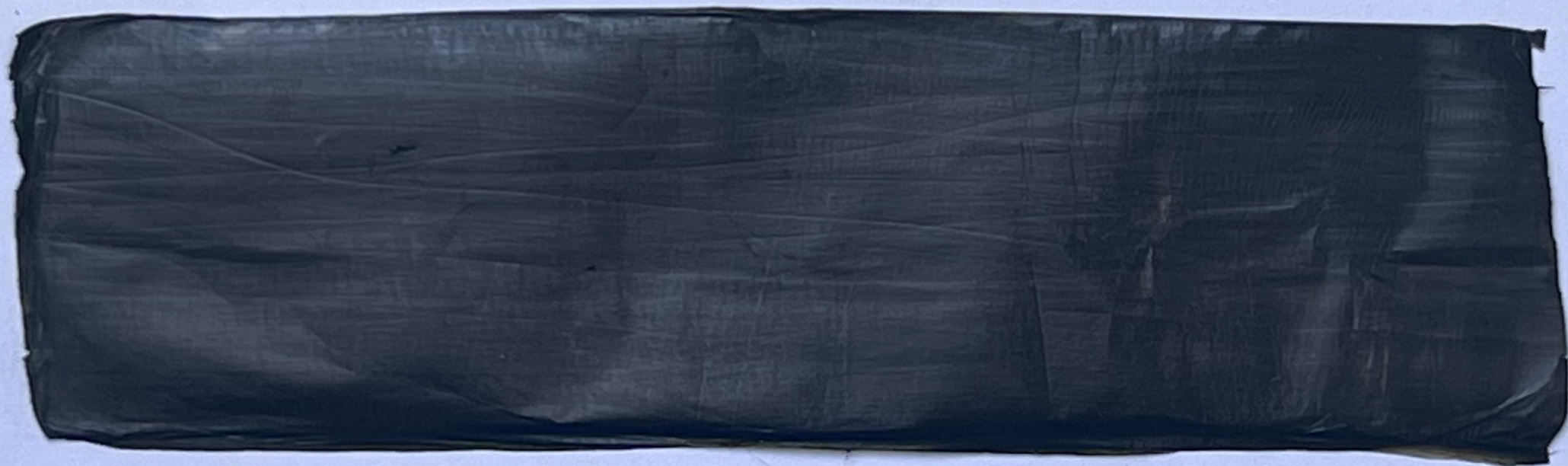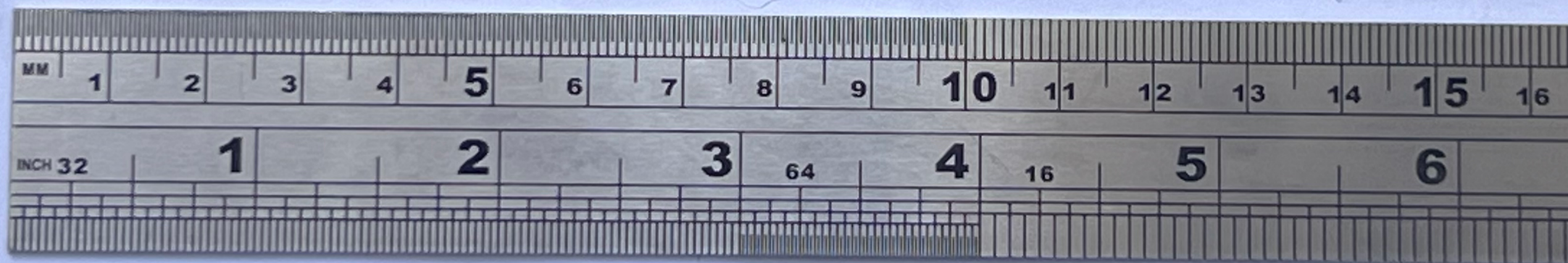

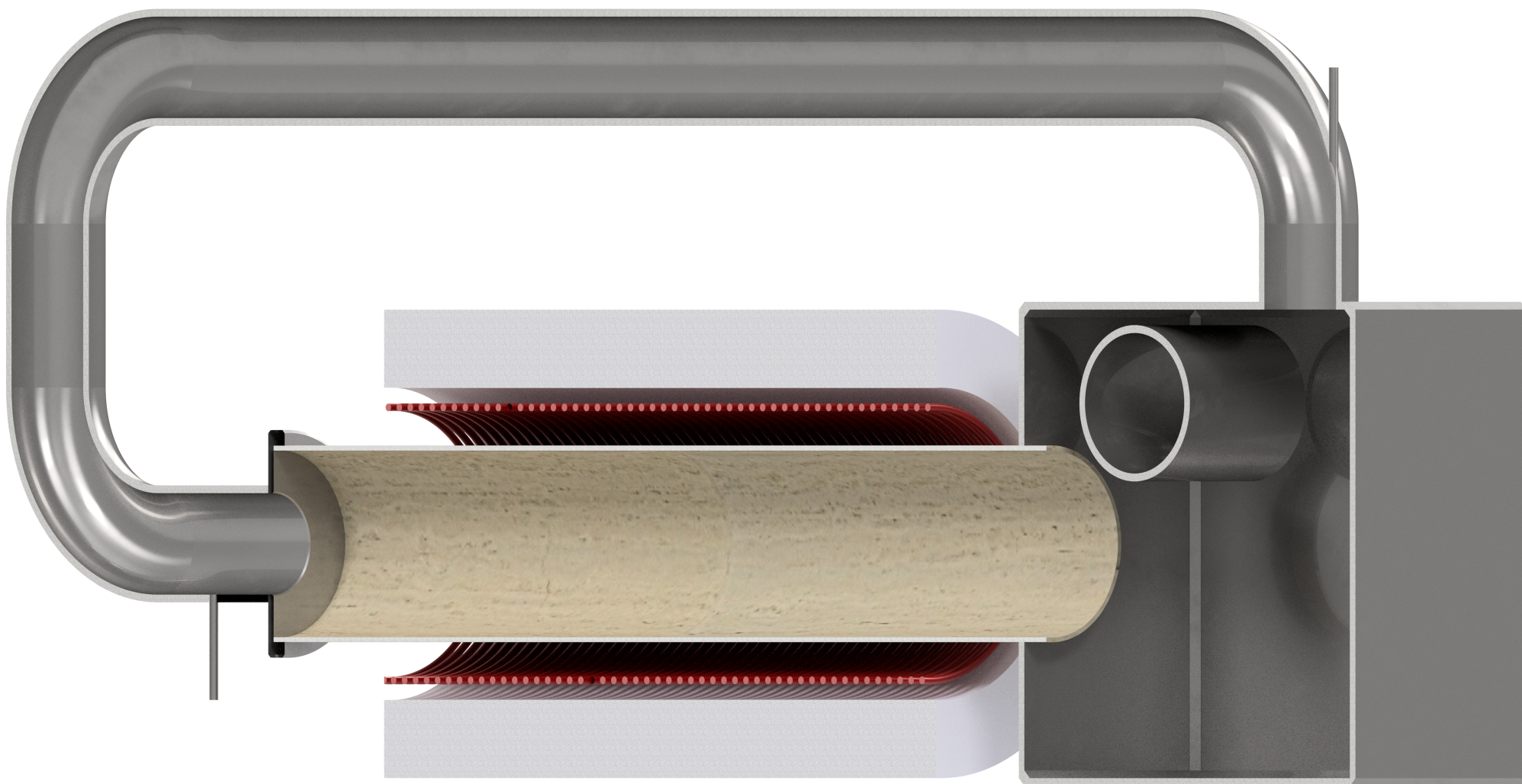

Supplement: Supplementary file 5 — Source_Data_Figure_2_Images.pdf contains unprocessed images of the aerogel and mat used in Fig. 2b,c, and unprocessed render of the reactor used in Fig. 2a. Source_Data_Figure_2.xlsx contains the H2 concentrations and effluent flows used to calculate the average H2 concentration and effluent flow in Fig. 2a; data used to calculate the product, waste and recycle stream proportions in Fig. 2d,e. [file 41560_2025_1925_MOESM5_ESM.zip › Source_Data_Figure_2/Source_Data_Figure_2_Images.pdf]

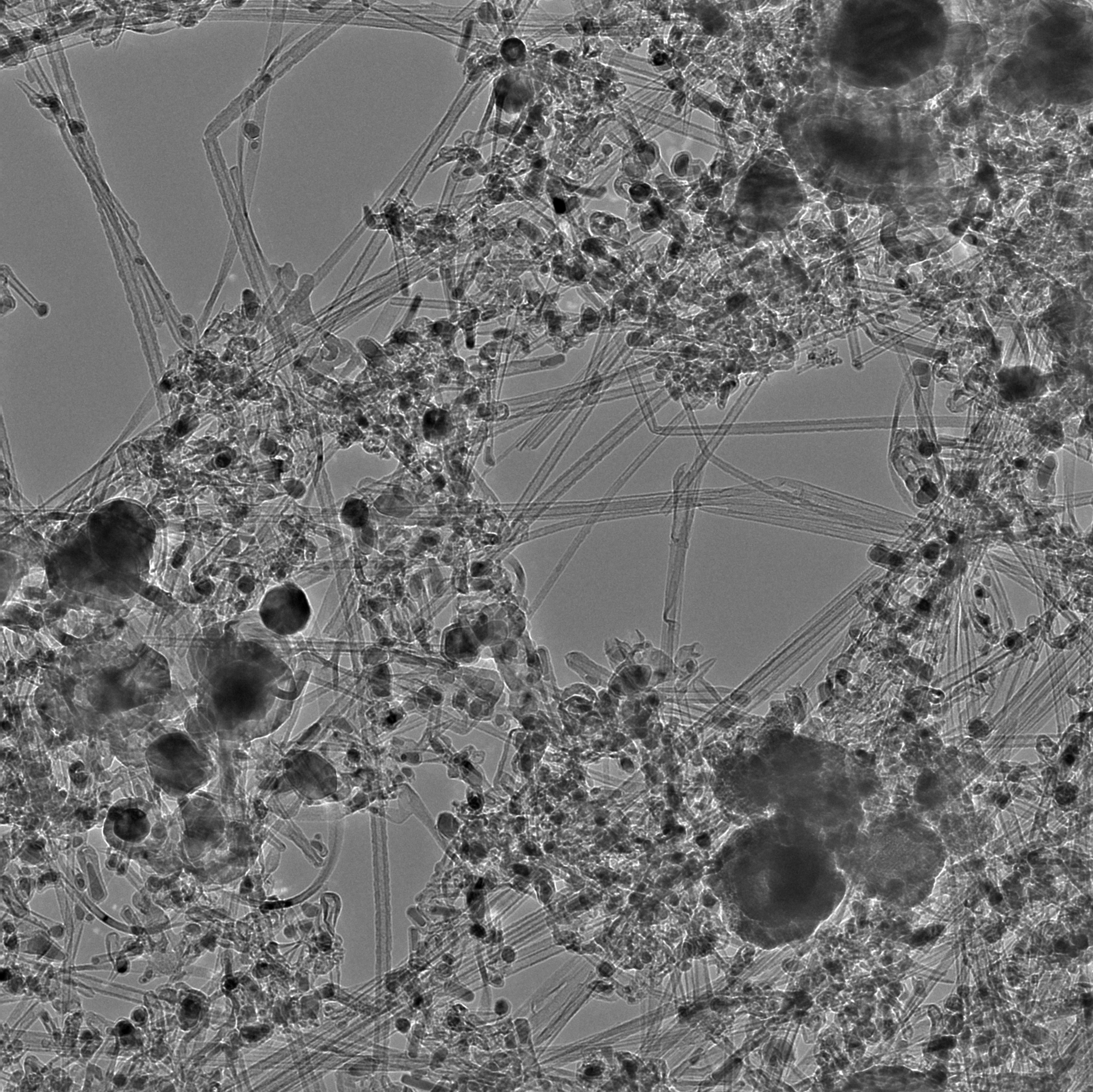

— 200 nm

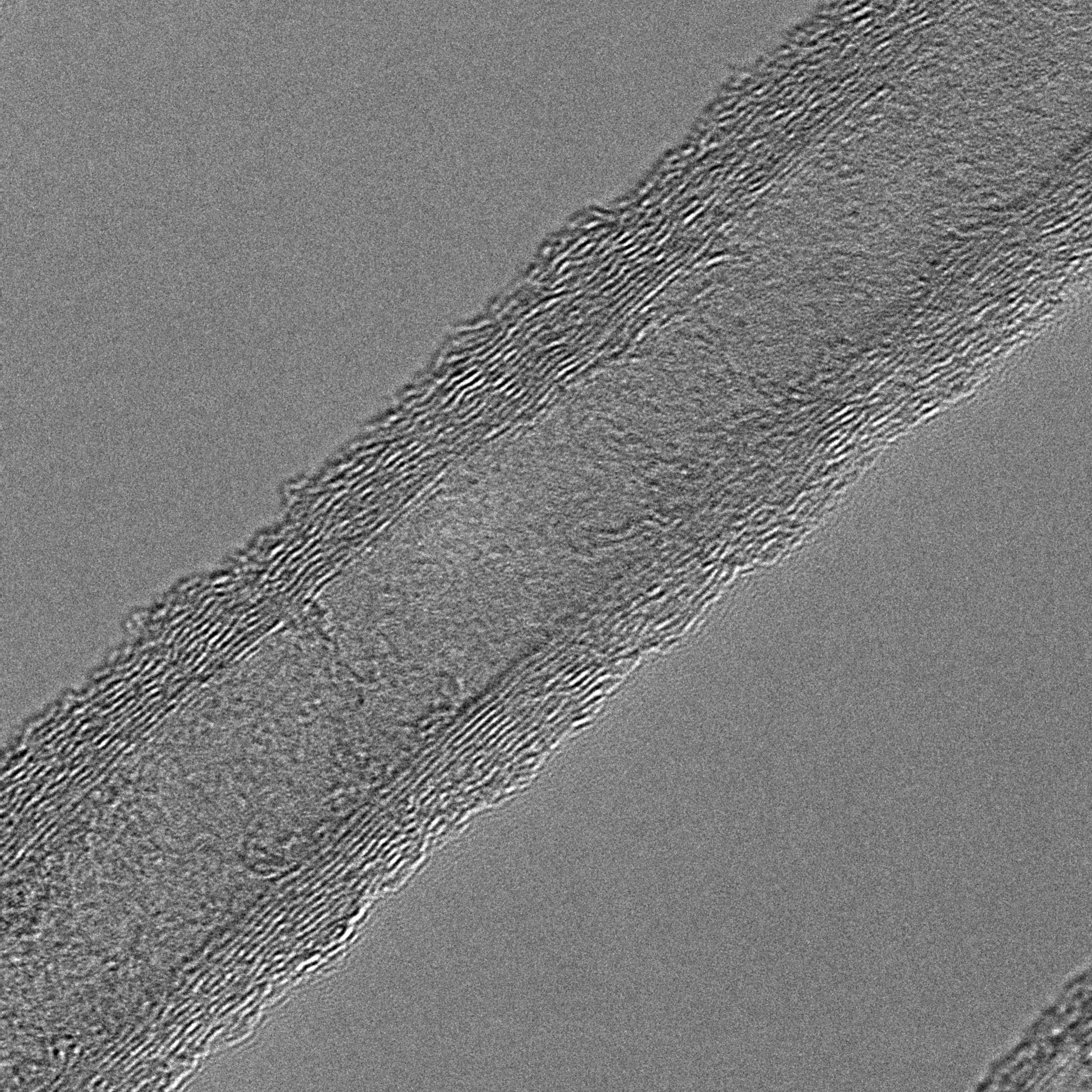

10 nm

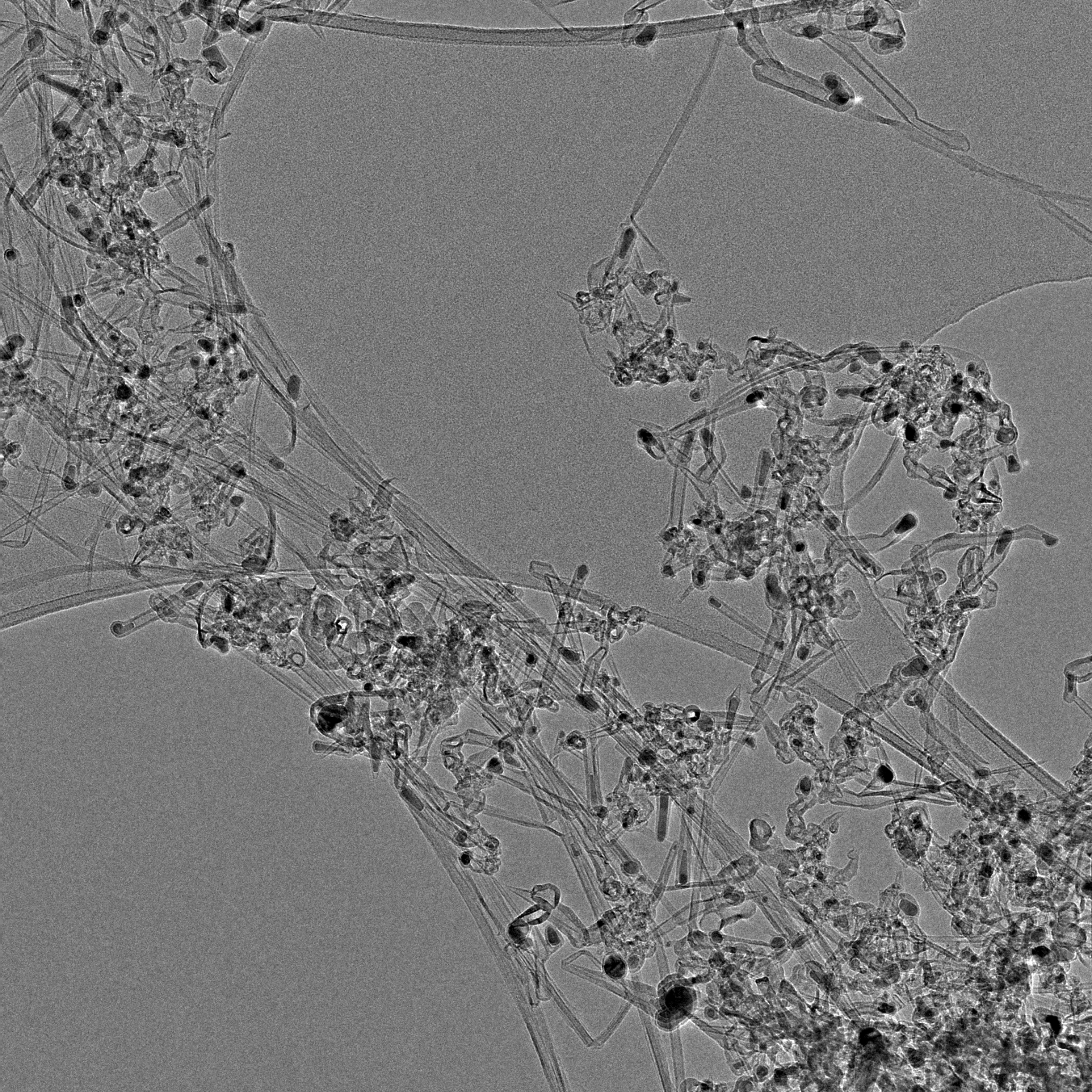

— 200 nm

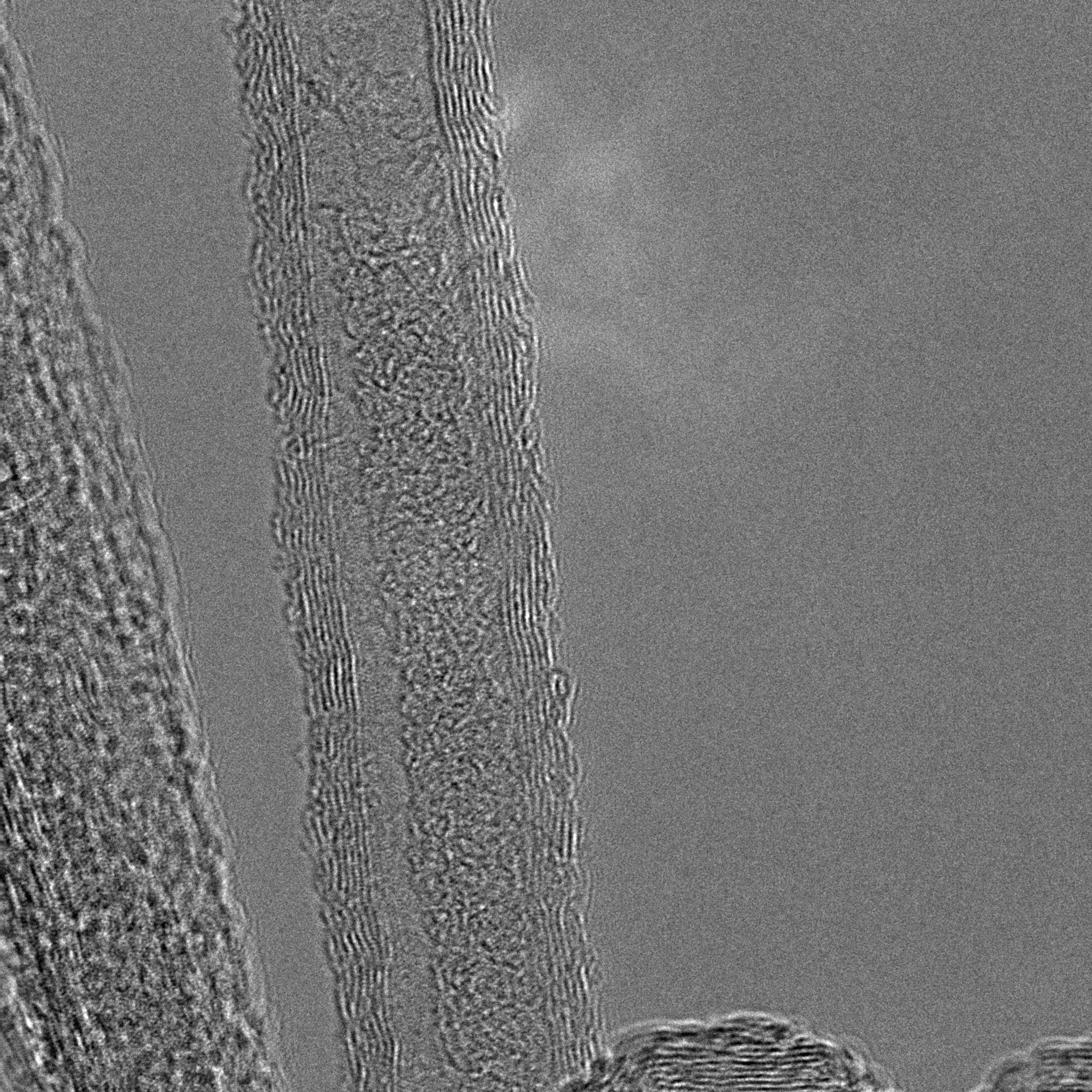

10 nm

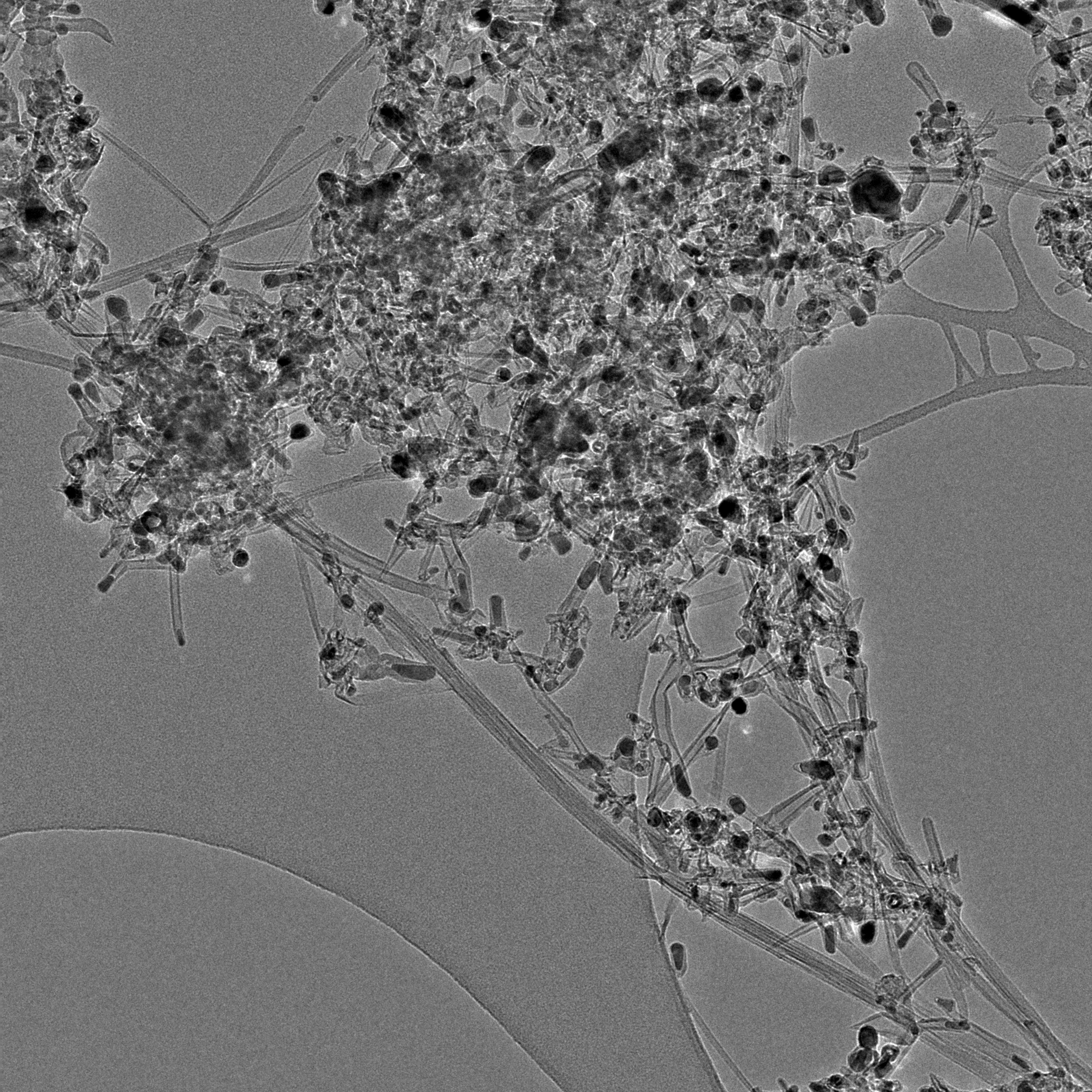

— 200 nm

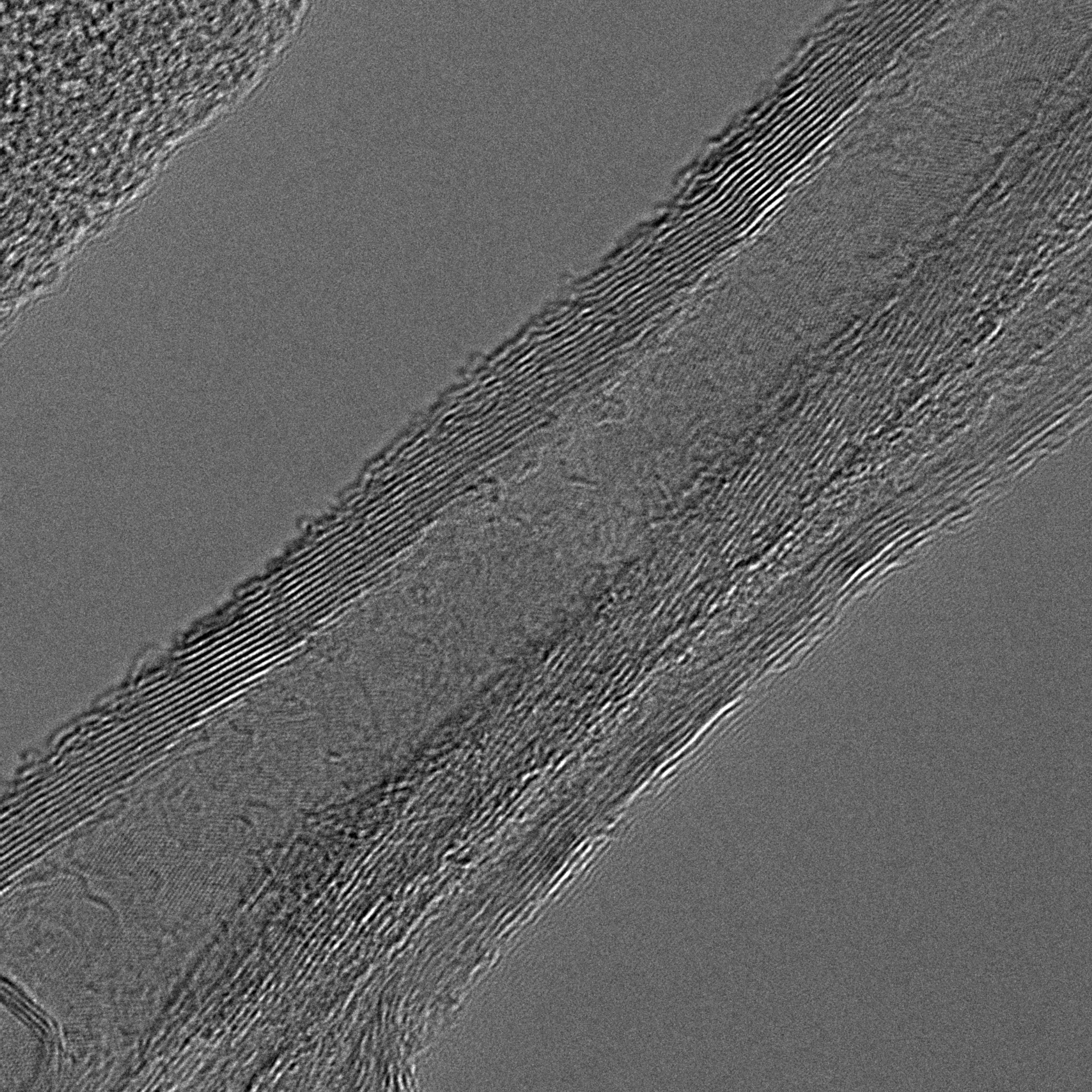

10 nm

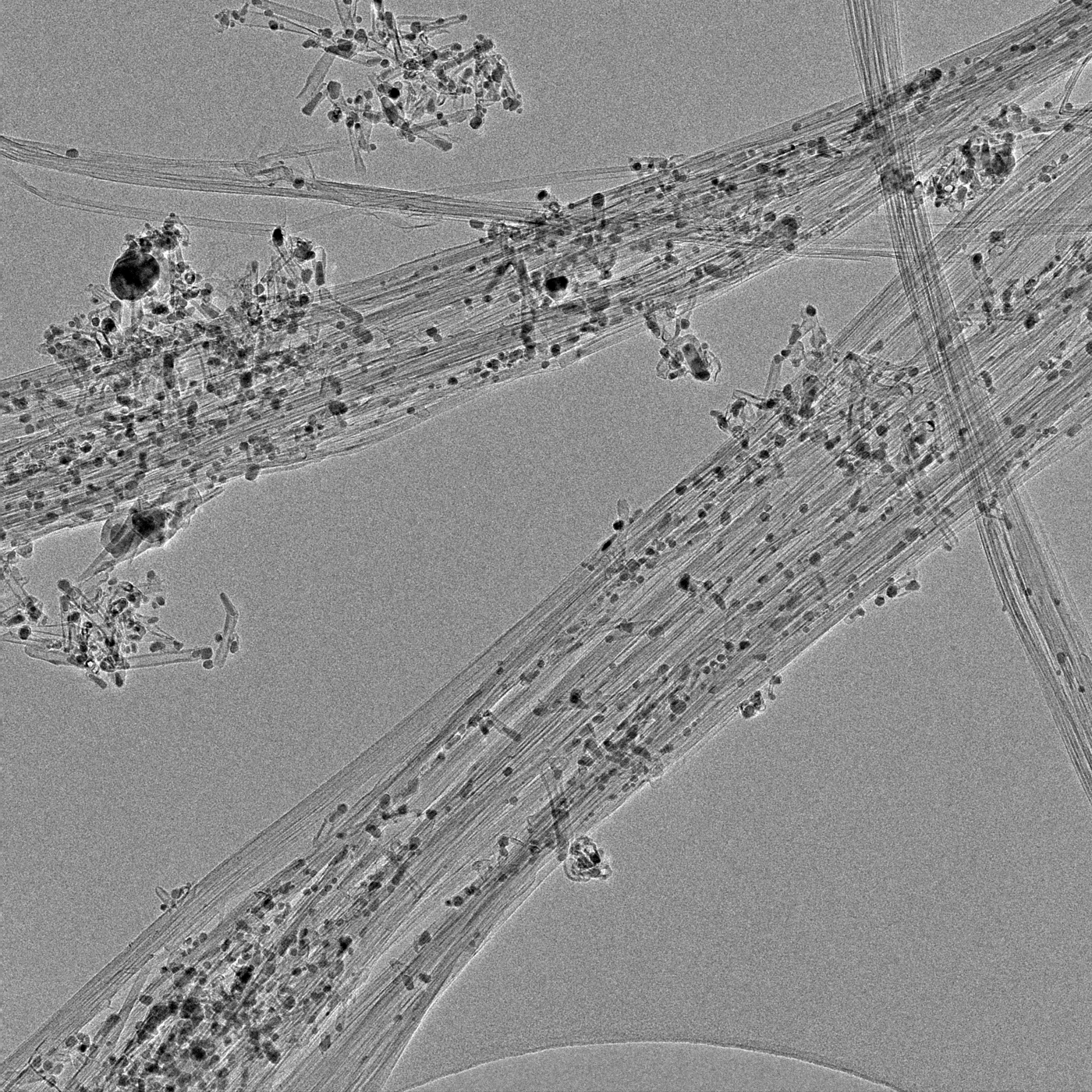

200 nm

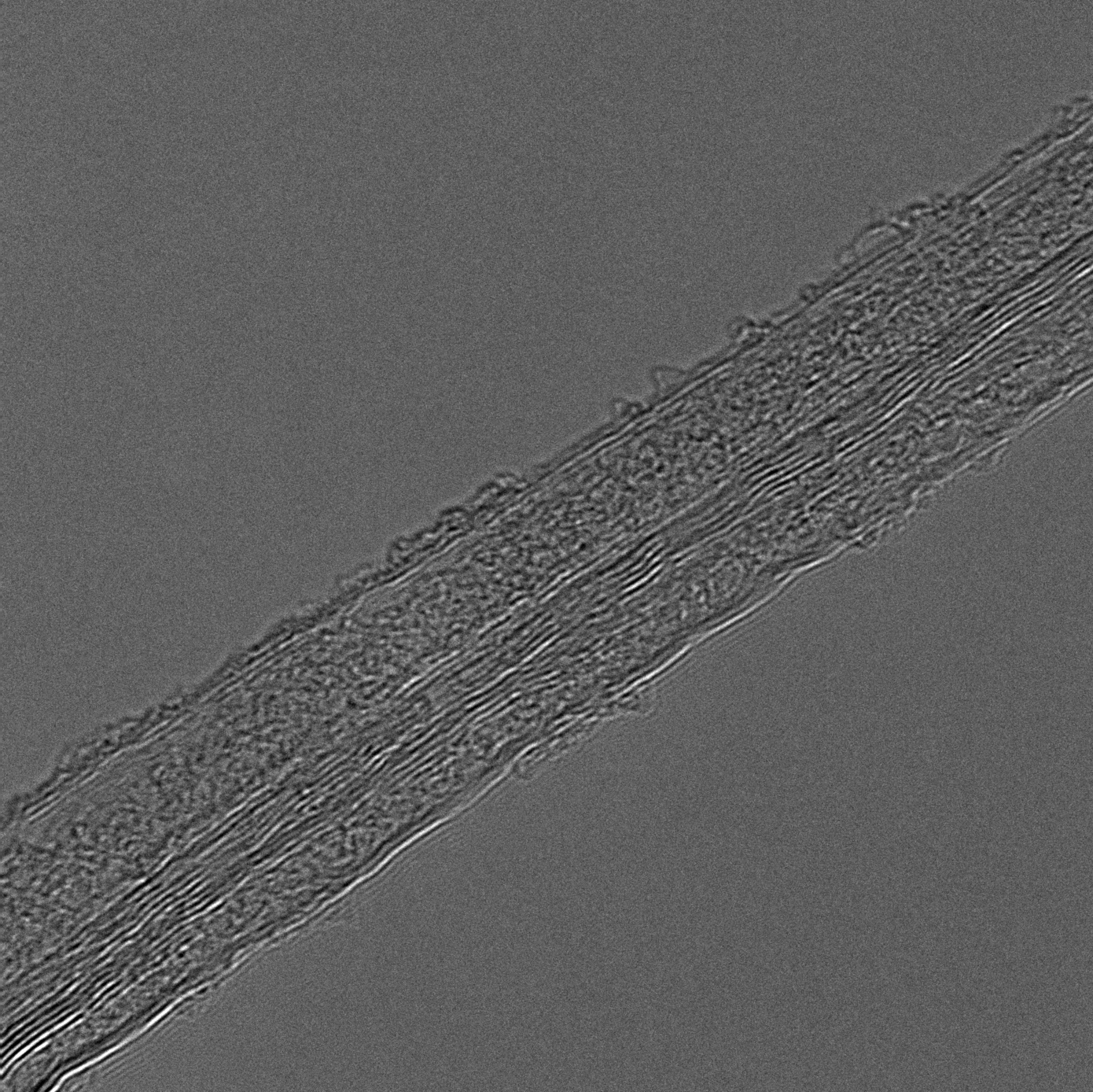

10 nm

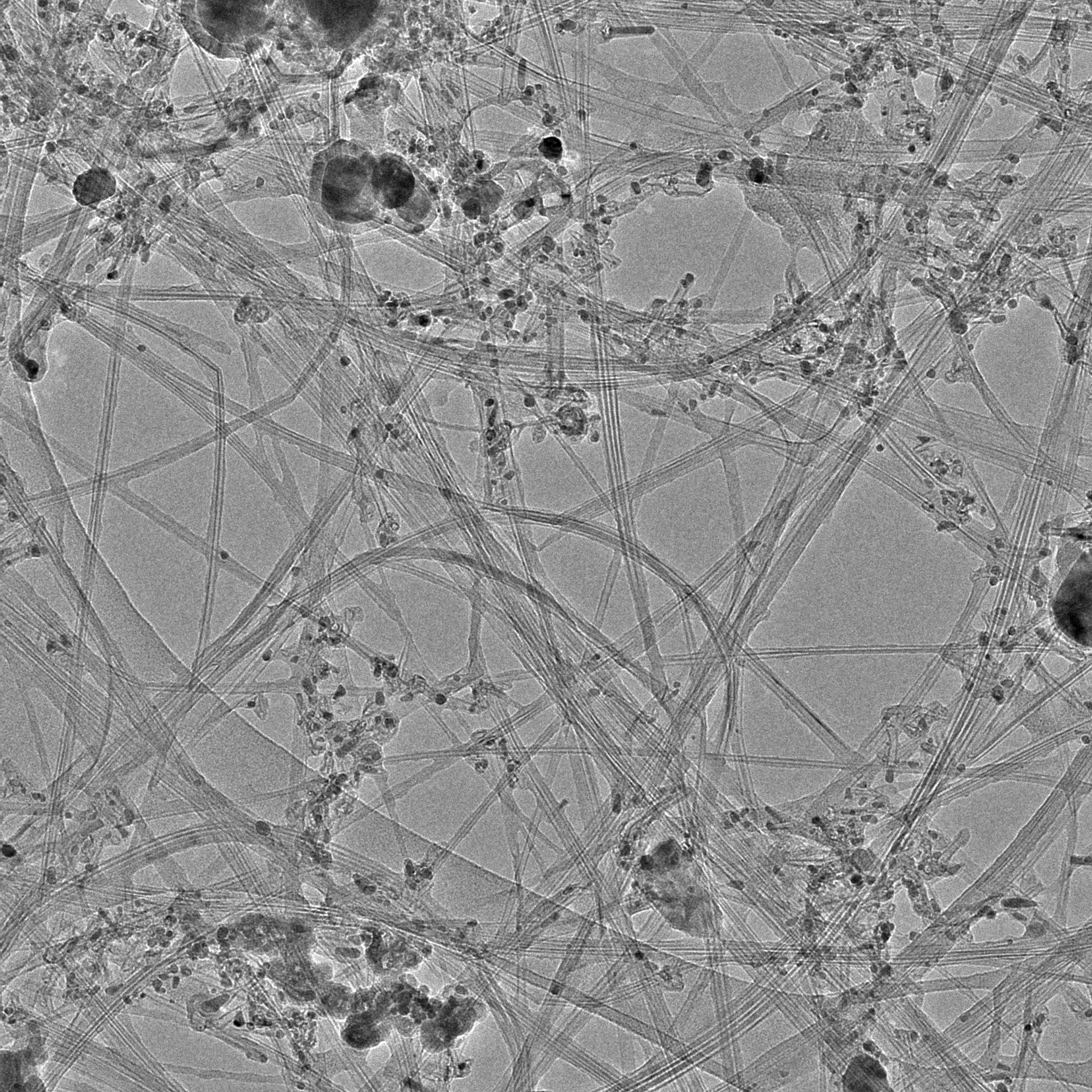

— 200 nm

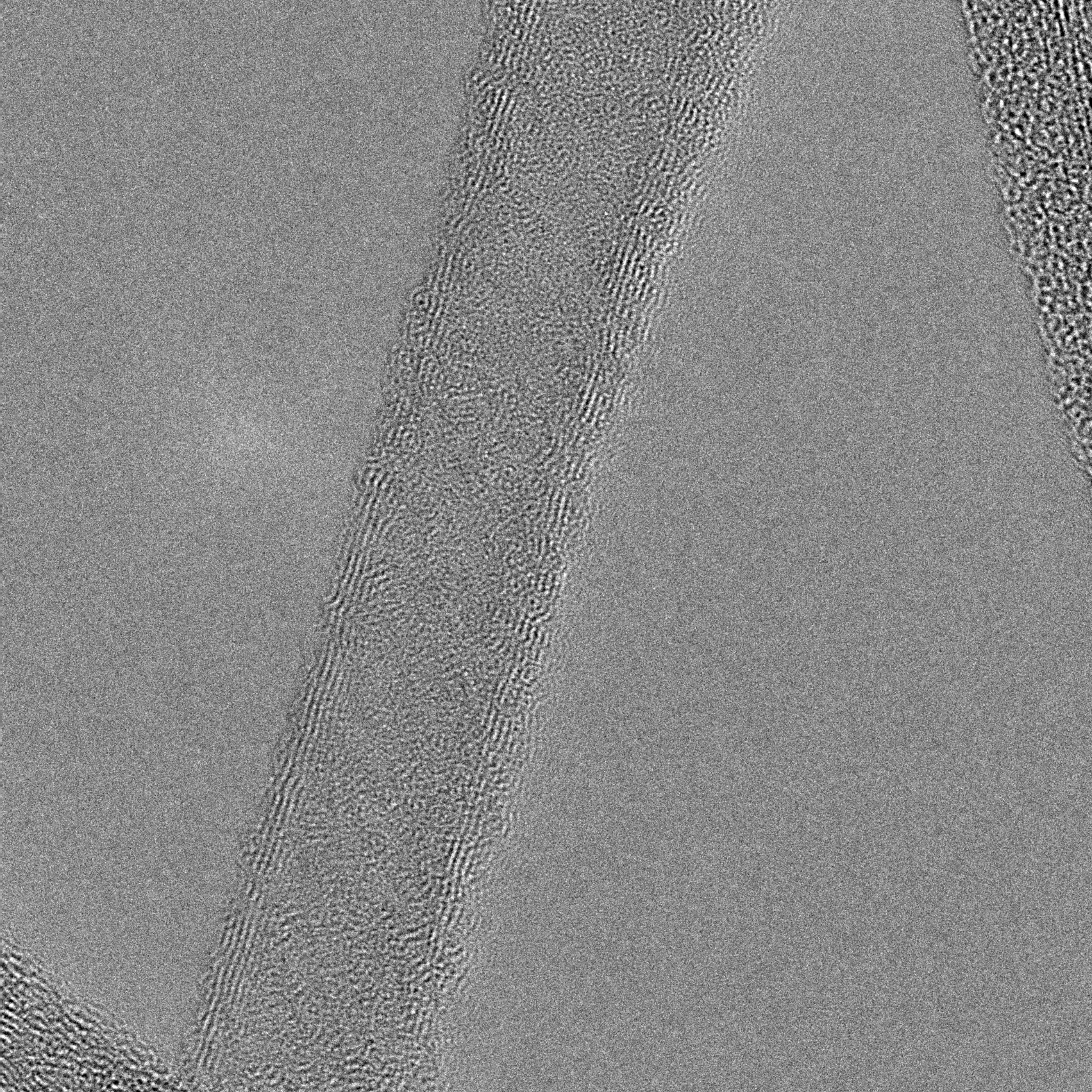

10 nm

Supplement: Supplementary file 7 — Source_Data_Figure_5.xlsx contains the data for each repeat used to obtain the average values presented in. Fig. 5a. Source_Data_Figure_5_images.pdf contains the unprocessed TEM images used in Fig. 5d–h. [file 41560_2025_1925_MOESM7_ESM.zip › Source_Data_FIgure_5/Source_Data_Figure_5_images.pdf]
